# Fuzzy technique for microcalcifications clustering in digital mammograms

**DOI:** 10.1186/1471-2342-14-23

**Published:** 2014-06-24

**Authors:** Letizia Vivona, Donato Cascio, Francesco Fauci, Giuseppe Raso

**Affiliations:** 1 Dipartimento di Fisica e Chimica, Università Degli Studi di Palermo, Palermo, Italy

**Keywords:** Breast cancer, Microcalcifications, Spatial filters, Clustering, Fuzzy logic, C-means, Mammography, Segmentation

## Abstract

**Background:**

Mammography has established itself as the most efficient technique for the identification of the pathological breast lesions. Among the various types of lesions, microcalcifications are the most difficult to identify since they are quite small (0.1-1.0 mm) and often poorly contrasted against an images background. Within this context, the Computer Aided Detection (CAD) systems could turn out to be very useful in breast cancer control.

**Methods:**

In this paper we present a potentially powerful microcalcifications cluster enhancement method applicable to digital mammograms. The segmentation phase employs a form filter, obtained from LoG filter, to overcome the dependence from target dimensions and to optimize the recognition efficiency. A clustering method, based on a Fuzzy C-means (FCM), has been developed. The described method, Fuzzy C-means with Features (FCM-WF), was tested on simulated clusters of microcalcifications, implying that the location of the cluster within the breast and the exact number of microcalcifications are known.

The proposed method has been also tested on a set of images from the mini-Mammographic database provided by Mammographic Image Analysis Society (MIAS) publicly available.

**Results:**

The comparison between FCM-WF and standard FCM algorithms, applied on both databases, shows that the former produces better microcalcifications associations for clustering than the latter: with respect to the private and the public database we had a performance improvement of 10% and 5% with regard to the *Merit Figure* and a 22% and a 10% of reduction of false positives potentially identified in the images, both to the benefit of the FCM-WF. The method was also evaluated in terms of Sensitivity (93% and 82%), Accuracy (95% and 94%), FP/image (4% for both database) and Precision (62% and 65%).

**Conclusions:**

Thanks to the private database and to the informations contained in it regarding every single microcalcification, we tested the developed clustering method with great accuracy. In particular we verified that 70% of the injected clusters of the private database remained unaffected if the reconstruction is performed with the FCM-WF. Testing the method on the MIAS databases allowed also to verify the segmentation properties of the algorithm, showing that 80% of pathological clusters remained unaffected.

## Background

Breast cancer is the most common cancer affecting women worldwide. It is visible in two forms: microcalcifications (small calcium deposits appearing as small bright dots on the mammogram) and massive lesions. These forms exhibit different typical characteristics, such as density, size, shape and number. For this reason, the algorithms implemented to detect both types of lesions have to be different.

The main difficulties for radiologists to detect microcalcifications are due to their small size (0.1–1 mm, mean diameter ~ 0.3 mm) and low contrast compared to the background of the images. In this context, a CAD system may help physicians to improve their performance [1-3]: a good CAD system must be able to suppress the noise in the image in order to improve the contrast between the Region Of Interest (ROI hereafter) and the background, and extract/select the lesions for a correct classification process.

In a CAD system, an unidentified microcalcification at the initial phase of image processing is considered completely lost, with severe limitations for later stages; for this reason several filtering techniques are applied to reduce the signal to noise ratio (SNR) [4]. But an equally important role is carried out by the classification phase, where training based procedures such as artificial neural networks are used, and a feature reference pattern set is required in order to correctly assign an unknown pattern, related to a lesion or to a particular membership class [5-9].

The clusters of microcalcifications are an important warning sign for breast cancer and they are present in 30 - 50% of screening mammography cases [10], therefore, their identification plays an important role in the phase image processing. The clustering is a type of classification imposed on a finite set of objects, characterized as points in a d-dimensional metric space in which every dimension corresponds to a feature (e.g. color, size, shape, position) [11].

Many techniques may be used for microcalcifications clustering: some are based on the simple Euclidean distance evaluation, others are related to the most significant features.

The procedure described by Nishikawa *et al*. [12] was applied to signals defined by a single pixel: signals with several pixels in area are reduced to single pixel by means of a recursive transformation. The number of signals within a small region, typically 3.2 mm × 3.2 mm, are counted: only if three or more signals are present within such a region, they are preserved in the output image. 78 mammograms were examined containing 41 clusters and a reduction in the false positives rate detection from 4.2 to 2.5 per image was found, while maintaining a sensitivity of approximately 85%.

Estevez *et al*. [13] proposed an algorithm (interactive selective and adaptive clustering, ISAAC) for assisting the radiologist in looking for small clusters of microcalcifications. This algorithm can be divided into two successive steps: selective clustering and interactive adaptation. The first step reduces the false positives number by identifying the microcalcifications subspace in the feature space; the second step allows the radiologist to improve results by identifying interactively additional false positive or true negative samples. The algorithm was tested on a 15 mammograms database. Performance of the method have been evaluated not by numerical parameter but by asking to three radiologists to determine, on a scale of 1 to 10 how helpful the method was in locating suspicious microcalcifications cluster areas, and by valuing capability of two other radiologists of identifying clusters observing the mammograms before and after the ISAAC application.

Mao *et al*. [14] proposed a distance-based and dense-to-sparse grouping method: the basic idea is to group the microcalcifications close enough to each other by examining the distance among them. The most closely distributed regions can be grouped into clusters first and relatively more widely distributed regions can be gradually grouped from evident cluster centers if they are still near enough to the centers. Several experiments were performed on a set of 30 mammograms containing 40 micro-calcification clusters. The method yields a result independent of the distribution orientation of clusters.

Arodz *et al*. [15] performed a sequence of morphological operations on the filtered image with the goal of eliminating small or isolated objects: an area opening operation (removal of objects smaller than threshold), followed by dilation (for removing isolated objects). The technique was applied to 50 mammograms, each of them showing a region of the breast with an area of 25 cm2 containing a suspicious lesion, and displayed higher efficiency levels for those clusters with a probability of 40% to be malignant. Performance of the method was evaluated by valuing the average number of clusters detected on mammograms processed by the system but not detected on original mammograms and by valuing the average estimate of detection improvement resulting from using the system.

Cihan *et al*. [16] used the subtractive clustering; this subtractive clustering is a fast one-pass algorithm for estimating the number of clusters and the cluster centers in a dataset, if no prior knowledge of number of clusters is available. The point with the highest number of neighbours may be a center point and is selected as the first cluster. The method has been applied to 34 mammograms with a total of 72 micro-calcification clusters. The results show a success rate of 93% for the proposed algorithm.

Riyahi-Alam *et al*. [17] proposed an automated segmentation of suspicious clustered microcalcifications on digital mammograms. The algorithm consists of three main processing steps for this purpose. In the first step, the improvement of the microcalcifications appearance by using the “a trous wavelet” transform which could enhance the high frequency content of breast images were performed. In the second step, individual microcalcifications were segmented using wavelet histogram analysis on overlapping subplanes. Then, the extracted histogram features for each subplane used as an input to a fuzzy rule-based classifier to identify subimages containing microcalcifications. In the third step, subtractive clustering was applied to assign individual microcalcifications to the closest cluster. Finally, features of each cluster were used as input to another fuzzy rule-based classifier to identify suspicious clusters. The results of the applied algorithm for 47 images containing 16 benign and 31 malignant biopsy cases showed a sensitivity of 87% and the average of 0.5 false positive clusters per image.

Cordella *et al*. [18] proposed a method based on a graph-theoretical cluster analysis for automatically finding clusters on mammographic images. The proposed method starts by describing with a graph all the microcalcifications detected by an automatic algorithm: the graph nodes correspond to microcalcifications, while the edges of the graph encode the spatial relationships between microcalcifications. Each micro-calcification is linked, by an edge, to all the other ones. The weight of each edge is the Euclidean distance in the 2D space between the nodes connected by that edge. After such a graph is obtained, the GTC analysis is employed to remove all the tree edges with weights greater than a threshold value: in this way, the GTC method automatically groups vertices (microcalcifications) into clusters. Successively, clusters with less than three nodes are eliminated. The approach has been tested on a standard database of 40 mammographic images and turned out to be very effective even when the detection phase gives rise to several false positives. Performance of the method were measured in terms of Precision and Recall giving rise to a Precision value of 1 and a Recall value of 0.94.

Wang *et al*. [19] presented an approach based on fuzzy clustering to detect small lesions, such as microcalcifications and other masses, that are hard to recognize in breast cancer screening. A total of 180 mammograms were analyzed and classified by radiologists into three groups (n = 60 per group): those with microcalcifications; those with tumors; and those with no lesions. Analysis by fuzzy clustering achieved a mean accuracy of 99.7% compared with the radiologists’ findings.

Quintanilla-Dominguez *et al*. [20] presented a method for the automatic detection of microcalcifications implemented by feature extraction and sub-segmentation steps. The feature extraction step is improved using a top-hat transform such that microcalcifications can be highlighted. In a second step a sub-segmentation method based on the possibilistic fuzzy c-means clustering (PFCM) algorithm is applied in order to segment the images and as a way to identify the atypical pixels inside the regions of interest as the pixels representing microcalcifications. Once the pixels representing these objects have been identified, an ANN model is used to learn the relations between atypical pixels and microcalcifications, such that the model can be used for aid diagnosis, and a medical could determine if these regions of interest are benign or malignant. The classifier presented in this work has been tested on four different combination of features, obtained the following results: Sensitivity: 98.21%, 98.70%, 88.93%, 88.73%; Accuracy: 99.54%, 99.56%, 98.22%, 98.18%.

Malar *et al*. [21] proposed an approach for detection and classification of mammographic microcalcifications using wavelet analysis and Extreme Learning Machine (ELM). A total of 55 mammograms, including normal and microcalcifications images have been used, producing an Accuracy of 94%.

Cheng *et al*. [22] presented an approach to microcalcification detection based on fuzzy logic and scale space techniques (FLSS). First, they employ fuzzy entropy principal and fuzzy set theory to fuzzify the images. Then, they enhance the fuzzified image. Finally, scale-space and Laplacian-of-Gaussian filter techniques are used to detect the sizes and locations of microcalcifications. A dataset of 40 mammograms containing 105 clusters of microcalcifications is studied. Experimental results demonstrate that the proposed method can archive an accuracy greater than 97% with the FP rate of three clusters per image.

In the following paragraphs we will explain the algorithm and its application to a set of digital images. The findings will be subsequently presented.

## Materials and methods

According to the report of the radiologist, a cluster of microcalcifications must have precise geometrical and morphological requirements: a cluster is a set of localized microcalcifications, therefore a potential micro-calcification cannot be associated with a cluster that is spatially “distant”.

As a further observation, the spatial association of microcalcifications is a necessary but not sufficient condition for proper clustering: a good microcalcifications clustering must be able to gather objects not only spatially “near”, but also “near” from a point of view of the form and the visual information contained in them.

Moreover, another frequently encountered difficulty in developing clustering algorithms is the lack of knowledge of the number of clusters present in an image with microcalcifications. Within this context, this paper describes a method that aims to achieve two objectives: the first is to use a more powerful clustering process (FCM-WF) based on the standard FCM algorithm appropriately modified with the addition of some features; the second objective is to determine the optimal number of microcalcifications clusters that may be present in an image.

**Figure 1:**
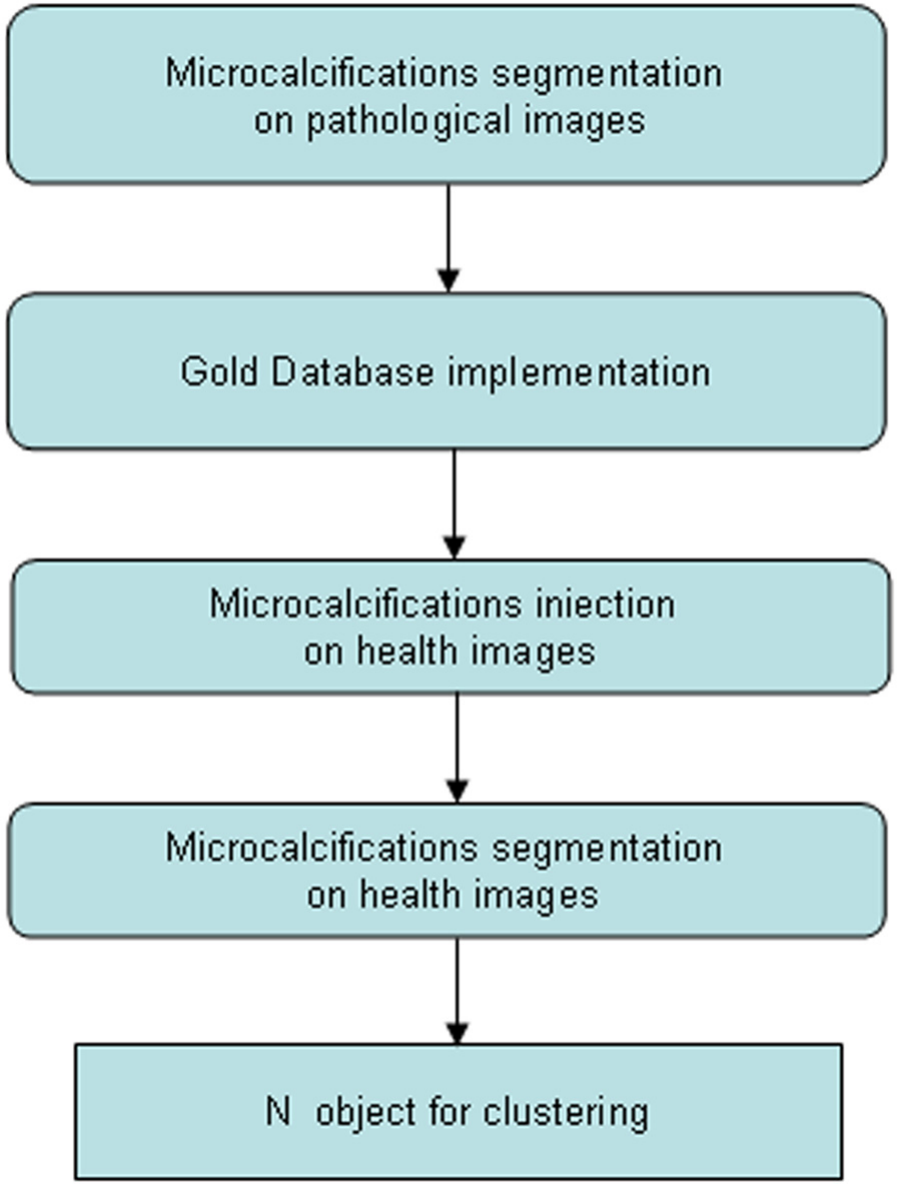
Procedure scheme.

In order to better evaluate the efficiency of the method, i.e. the correct identification of the cluster and the number of micro belonging to it, testing the algorithm on pathological images reported by the radiologists could not be sufficient because we need to know exactly both the correct position of the cluster and the number of microcalcifications belonging to the cluster. For this reason in this paper we have chosen to test the algorithm on images obtained by healthy images with an artificial injection of microcalcifications. The microcalcifications clusters used for simulation are extracted from real pathological images reported by several radiologists.The procedure followed in this preliminary phase is shown in Figure 1.

The first step of the procedure is the segmentation (Section Microcalcifications Segmentation), which is preliminary to other phases of the process as in all CAD system. Each cluster, with a number of micro variables depending on the lesions structure, is stored to build a database of real clusters (gold database). The mode of creating this "gold database" makes it possible to maintain in the cluster all relevant information present in the original image, with the consequent possibility to use them at a later stage (Section Databases). Afterwards, some clusters, or part of these, are injected on healthy images by creating a sample of pathological images "artificial" that will become our new "gold database artificial". Finally, with a new segmentation process performed on sample images, we obtain N objects used as a starting point to test the FCM-WF algorithm and to determine the number of clusters, as described in Section The Fuzzy C-MEANS Implemented With Features (FCM-WF) and Section K Clusters Best Value. The procedure was tested on images belonging to a private anonymous database collected in the Policlinic Hospital of Palermo. Policlinic Hospital is a hospital firm of University of Palermo in which formation, scientific research and health service are well integrated. Policlinic Hospital assess that every research involving human being is carried out in compliance with the Helsinki Declaration and correctly informing the patients previously. Policlinic Hospital of Palermo, in the person of Dr. Raffaele Ienzi who provided us the images, assessed that every precaution has been taken to protect the privacy of the patients and the confidentiality of their personal information. Only the images belonging to patients who given a free informed consent in writing have been used to create the database.

Anyway, since each paper described in the state-of-the-art is tested on different image databases and as such does not provide a perfectly fair comparison, in order to provide strong justification for the effectiveness of our work, we applied the FCM-WF algorithm even on the publicly available MIAS database (/http://peipa.essex.ac.uk/info/mias.html). The characteristics of this database will be described in Section The MIAS database.

## Microcalcifications Segmentation

In a CAD system, an important role is performed by the ROI extraction phase [23], because at this level a missed microcalcifications is definitely lost. The algorithms designed to enhance the contrast of microcalcifications may improve the ROI extraction performance [24].

It is possible to recognize microcalcifications within an image using mammography features edge detection [25]. The enhancement stage must be sensitive enough to emphasize low contrast objects while, at the same time, it must have the required specificity to suppress the background [26]. Usually the background corresponds to some smoothed regions of the image which don’t give relevant information about pathologies in many cases. Background suppression can be implemented by using high pass filtering. In order to detect contours, the Laplacian of Gaussian (LoG) is often used in practice; it combines the Gaussian filter with the search properties of the edges of the Laplacian. The filter equation is represented by:

(1) $$-\nabla^{2}H_{\sigma }\left(x,y\right)=-\frac{1}{\pi \sigma^{2}}\left(\frac{x^{2}+y^{2}-2\sigma^{2}}{2\sigma^{4}}\right)e^{-\frac{x^{2}+y^{2}}{2\sigma^{2}}}$$

The parameter σ of Gaussian controls the effect of the LoG: the rise of its value increases the smoothing effect but with the lost of the ability to discriminate the details; therefore it is closely correlated to the size of the objects to identify. In equation 1), standard deviation σ is related to the target of size [27]. The relationship between the parameter σ and the dimensions of the target to identify has as effect, for microcalcifications recognition, a variability in the ability of individualization of the filter inside the range of dimensions of microcalcifications. This dependence implies that, if the size of the object to identify varies as in our case, we must use a method of analysis that uses several forms of convolution, with different values of σ. Conversely, in a multiscale approach, the final result can derive from mean or from maximum value among different obtained results (different values of σ) but in both cases the same problems occur: in the first case the scales different from that with the best coupling between the value of σ and the size of the object have as a result the reduction of detection power; in the second case we have a high sensitivity to noise.

To overcome the dependence from target dimensions and to optimize the recognition efficiency, it has been necessary to study and implement a spatial filter with a form that allows the detection of small and large microcalcifications. This form-filter, designed with the sum of the weights equal to zero in order to get the effect on the derivative, is characterized by three regions:

1. a first central region, circular (of radius equal to R_1_), whose pixels have positive intensity, and area equal to the smallest size of a single micro, with the shape of the positive part of the Laplacian of Gaussian (LoG);

2. a second region, also circular, adjacent to the first region and outer, whose pixels have zero intensity, which extends up an area equal to the maximum size of a micro detectable (distance R_2_);

3. a third region, the most external and quite narrow, with negative values obtained from the same LoG function and renormalized so that the sum of the intensities of its pixels, negative, is equal to the sum of the intensity of the first region, positive.

By assuming ΔR = R_2_ - R_1_, mathematically the three regions of developed filter F(x, y) may be defined as follows:

$$F\left(x,y\right)=\left\{\begin{array}{lll} \text{Log}\left(x,y\right) & \text{where}: & x^{2}+y^{2}<R_{12^{2}}\\ 0 & \mathrm{where}: & R_{12^{2}}\leq x^{2}+y^{2}\leq R_{22^{2}}\\ \alpha \text{Log}\left[\left(x-\Delta R\right),\;\left(x-\Delta R\right)\right] & \text{where}: & x^{2}+y^{2}\geq R_{2^{2}} \end{array}\right)$$

where α is a normalization factor chosen such that the volumetric integral I_S1_, within the circle of radius R_1_, is equal to the volumetric integral I_S2_ of the surface outer to the circle of radius R_2_.

**Figure 2:**
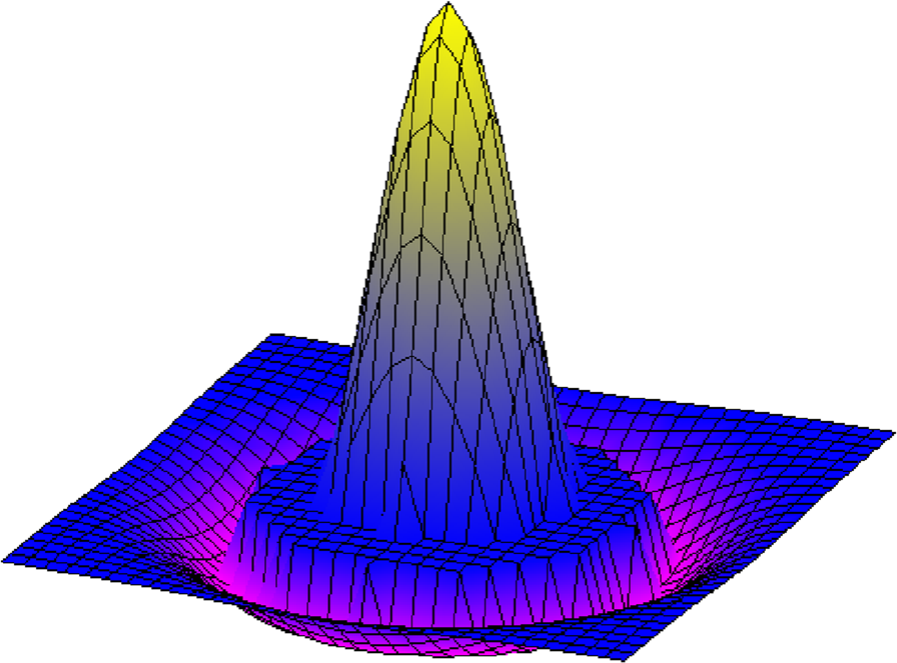
The Spatial filter implemented.

**Figure 3:**
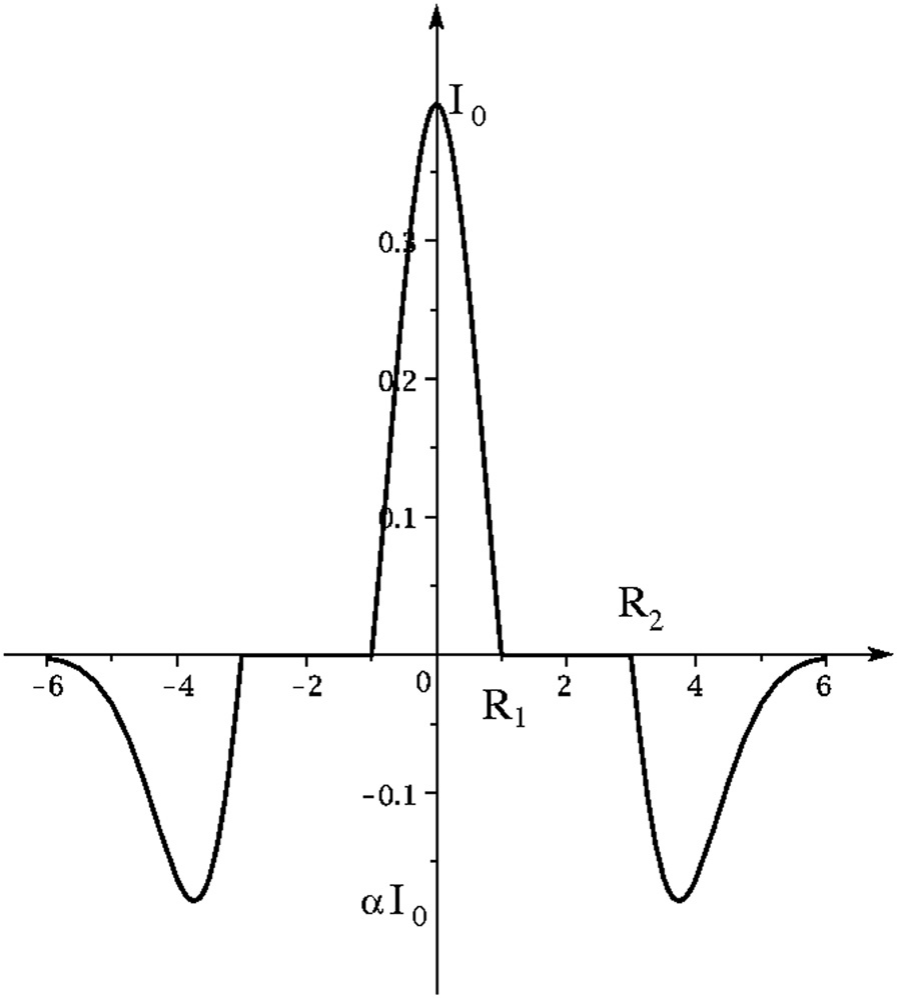
2D projection for the spatial filter.

**Figure 4:**
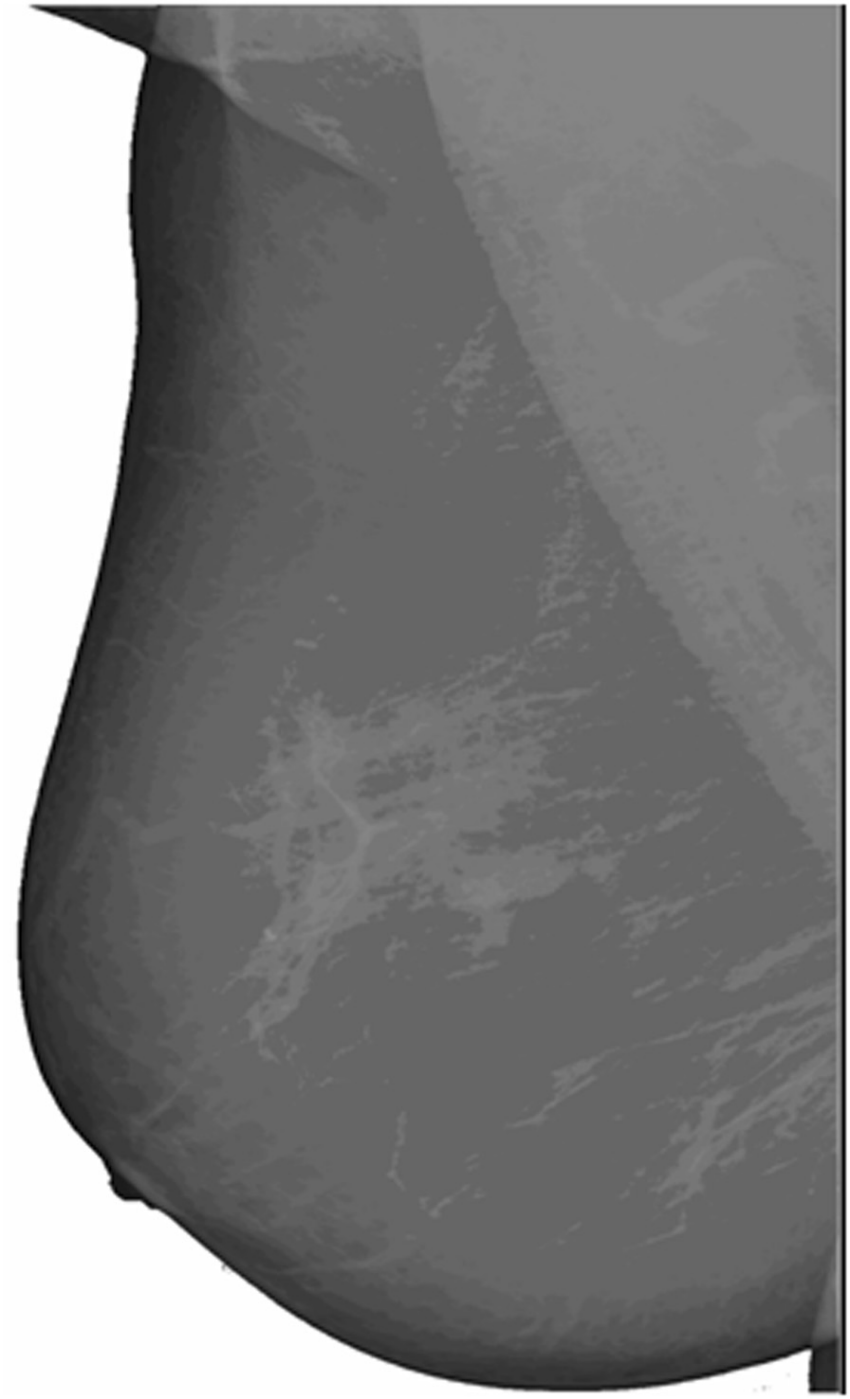
Digital mammography.

**Figure 5:**
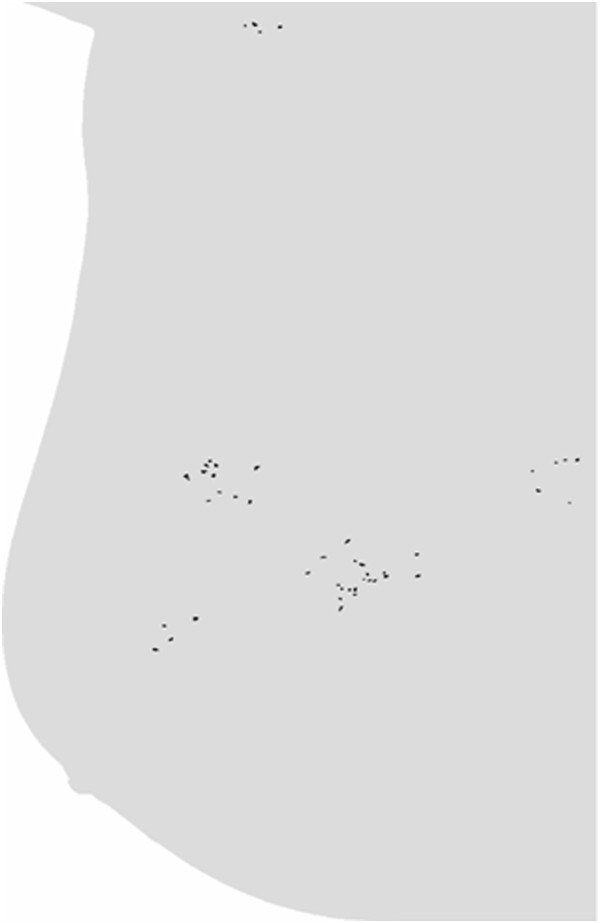
Filtered image.

From an analytic perspective, the filter function here realized is a LoG translated function: the negative LoG tail (that is: the third region of the developed filter) has been translated with ΔR, and the subsequent emptied region (the filter's second region, a circular crown of width ΔR) has been assigned a null value. Actually, the second region of the filter has the objective to make negligible the convolution contribution of the pixels characterized by distances between R1 and R2 and thus to obtain a constant detection performance on the pathology interval [R1, R2]; in fact, all the pixels of a ROI falling in the interval [R1, R2] and geometrically lying on a circular crown, will give no contribution in the computing of the convolution. So we can strongly reduce the dependence of the recognition process from the variability of the dimensions of microcalcifications. Since the aforementioned translation occurs in the plane and quadratically increases the number of interested pixels, a renormalization (performed by acting on the parameter α from the third region) becomes compulsory in order to fulfill the null sum condition (as required by the derivative filter definition). If one analyzes the LoG filter and the result of its application on a generic (x,y) position of an image, one notes that for obtaining a positive value in (x,y) the ROI must correspond to the positive part of the filter, while the background must overlap with the negative part. The filter developed here allows also partial overlaps of the ROI and the positive part of the filter. In fact, for ROI characterized by linear dimensions between R1 and R2, the positive part which will overlap with the filter still remains the part of linear dimensions R1.Figure 2 shows the 3-D representation of the filter, Figure 3 shows the 2-D projection and its renormalization due to the effect of placing the region with null values. In our case, application to a discrete image, the 3-D integrals are equivalent to the sum of the pixels intensities of the two affected areas, positive and negative, respectively.Figures 4 and 5 show how the filter implemented can emphasize the presence of microcalcifications: Figure 4 shows original mammograms in which microcalcifications clusters are hidden by breast tissues; Figure 5 shows the microcalcifications that remain after the segmentation process: in this figure it is possible to recognize the five clusters hidden in Figure 4. The image colours are inverted for better visualization.

## Databases

### Gold Database Implementation and Injection Process

A medical image data set is the starting point for important epidemiological and statistical studies. Usually, such a set is used to develop and test algorithms for computer aided detection (CAD) systems but it is used also for teaching and training medical students or as an archive of cases. The development of a CAD system is intimately linked to collection of a dataset of selected images [28]. The purpose of this step was the creation of a gold database of mammographic images [29] containing microcalcification clusters to be used for the evaluation of the clustering process. We used a private database because we need to know all the truth and the details of microcalcifications clusters, i.e. not only the healthy or pathological nature of microcalcifications, but even all the useful properties like the mean intensity over the background, the number and the position inside a cluster, the geometrical and spatial distribution of clusters on breast tissue and so on.

Two expert radiologists took place to the segmentation process: after the comparison, they segmented semi-automatically the ROI they though being microcalcifications. The health microcalcifications have been confirmed by a 2 years follow up and the pathological ones with histological exam.

Among all the ROI extracted by segmentation process, only those “cluster of ROI” identified by the physician as pathological cluster are kept to constitute the gold database of microcalcifications, while all other ROI are discarded. For all the microcalcification of each cluster we have recorded: mean intensity, number and coordinates of pixels, and membership cluster.

Since the properties that make possible to classify a group of microcalcifications as a cluster certainly take into account the parameters of size, intensity over the background, relative distances of the microcalcifications and cluster geometry itself, each simulated cluster must retain all informations of original cluster. From these data, we injected the cluster of microcalcifications in healthy images according to the following steps:

1. random extraction of one microcalcification from a cluster (mother cluster);

2. random selection of a position within the breast and injection of the extracted microcalcification;

3. random extraction of a new microcalcification from the same “mother cluster” and its injection on the breast retaining angles and distance with the previous microcalcification.

Step 3 is repeated until injection of a microcalcification number n in the range [P,M], where M is the micro number within the mother cluster and P is the nearest integer to M*0.8 (20% less), with condition M ≥ 4.

To not interfere with the healthy breast tissue, this injection method takes into account the average intensity of each cluster related to the intensity of the parenchyma where the cluster is injected.

At the end of the injection process of microcalcification, the healthy image is segmented with the same method used for the pathological images. The number N of objects segmented will be used for the following step, that is the clustering algorithm.

### The MIAS database

**Figure 6:**
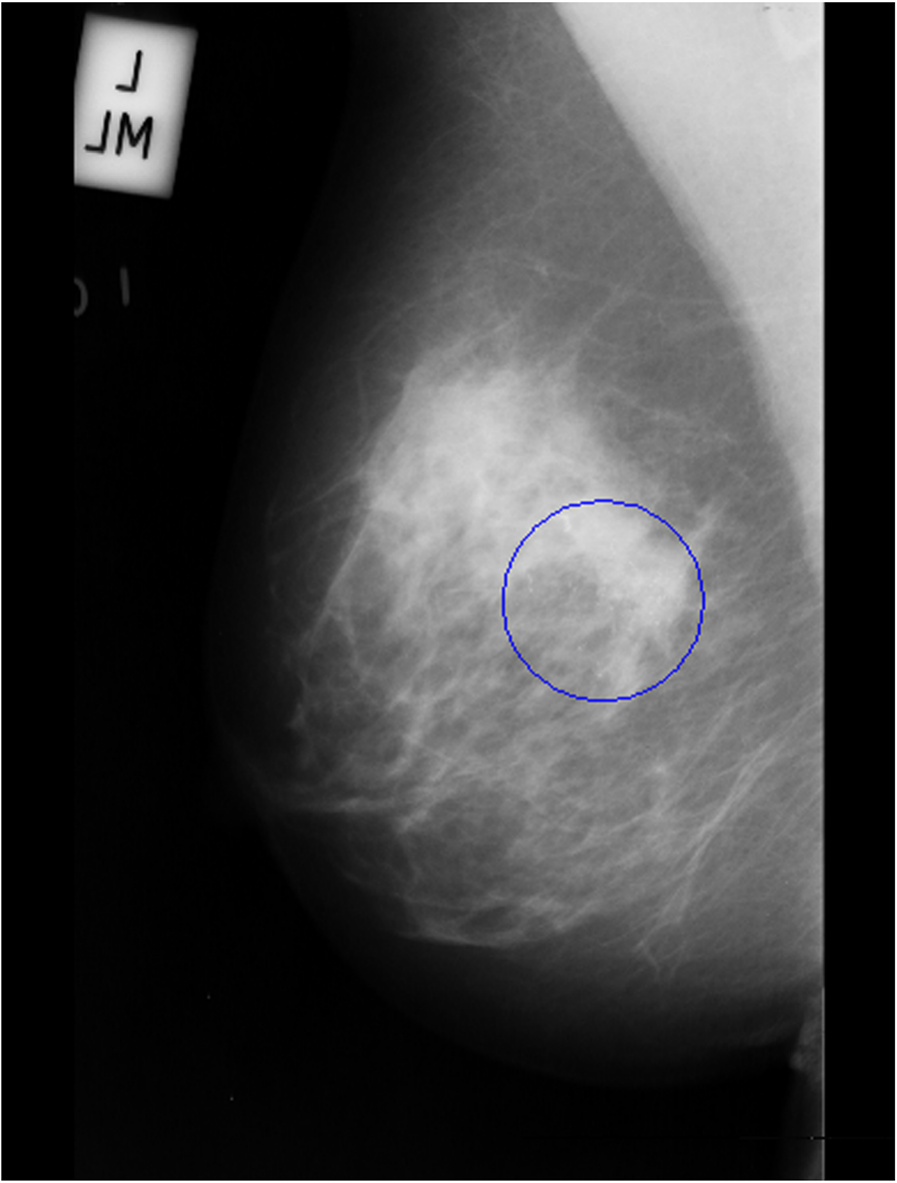
Original mammogram (im. mdb209).

**Figure 7:**
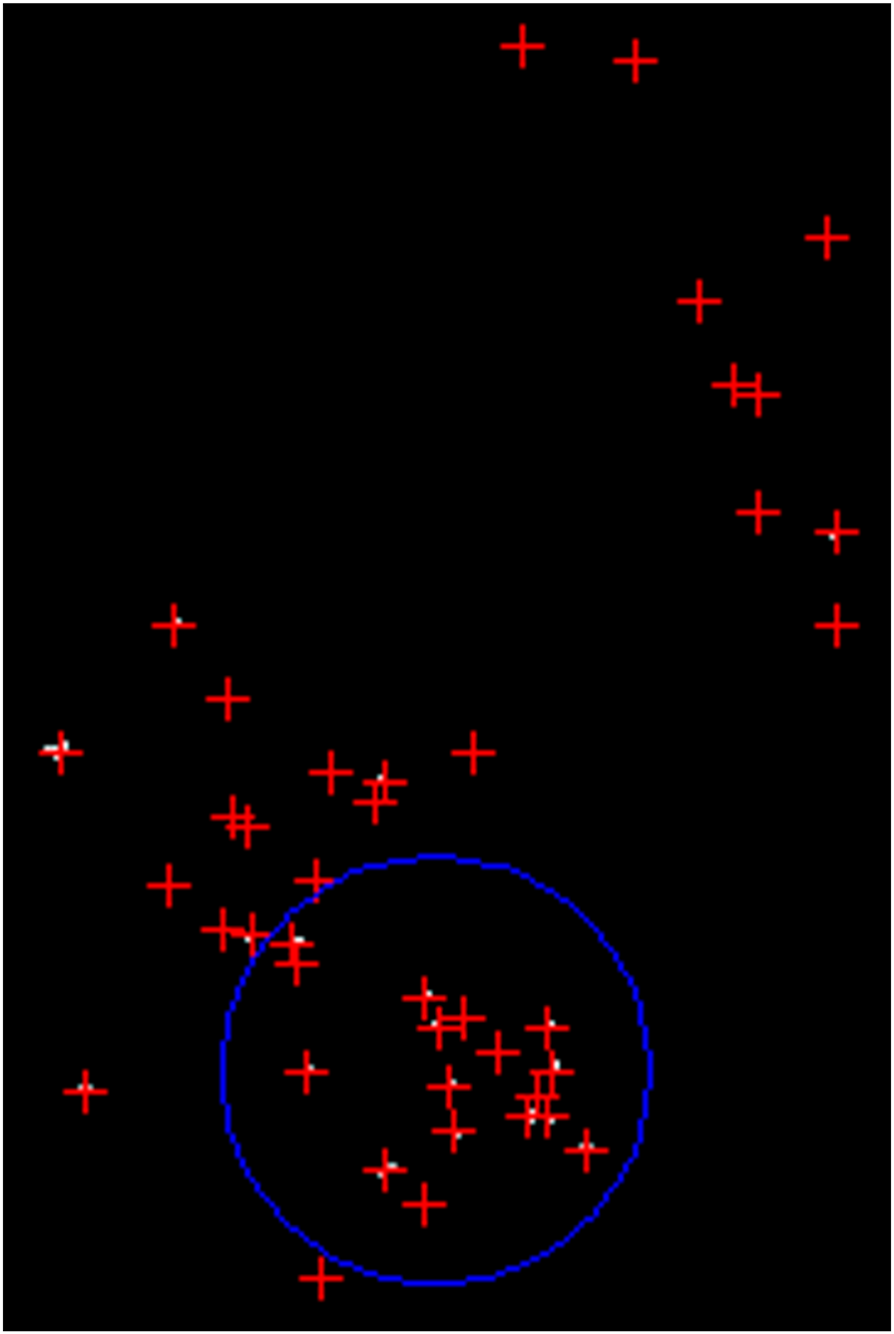
Mammogram after segmentation process.

The images collected from the Mammographic Image Analysis Society (MIAS), an organization of research groups in UK, are available via the Pilot European Image Processing Archive (PEIPA) at the University of Essex. The database contains 322 digitized films of 161 subjects with both right and left breast images and it also includes the so called ground truth on the locations of any abnormalities that may be present [21].To test the method we have to know position and number of microcalcifications belonging to the clusters, so among all the images contained in this database only the 20 images in which centre locations and radii of clusters are known have been used. Particularly, since the database provide only the centre locations and radii of clusters, and we need to know even position and number of every microcalcification belonging to it, we considered as “truth” about pathological microcalcifications the objects found inside the indicated circle after the segmentation process. An example is shown in Figure 6 and in Figure 7: Figure 6 shows the original mammogram (image mdb209), Figure 7 shows the same mammogram after the segmentation process, in which the centroids of all the segmented objects are indicated. In both figures the circle of cluster is present. We considered as microcalcifications belonging to the cluster only the objects found in the circle.

The images of MIAS database used contain one cluster, except images mdb223, mdb239, mdb249 (two clusters) and image mdb227 (three clusters), for a total of 25 clusters.

## The Fuzzy C-MEANS Implemented With Features (FCM-WF)

As already explained in Paragraph 1, clusters of microcalcifications are an important warning sign for breast cancer so their identification plays an important role in developing a CAD system. Anyway, detecting all microcalcifications clusters is not an easy task, as there is often poor contrast on mammograms between microcalcifications and the surrounding tissue. Since microcalcifications belonging to the same cluster have similar properties (or *features*) such as grey level intensity, the main objective of a clustering process is to evaluate this similarity according to a distance measure between the microcalcifications and the prototypes of groups, and each microcalcification is assigned to the group with the nearest or most similar prototype [20].

Several algorithms should be proposed to solve the problem of microcalcifications clustering. Among them, partitional clustering methods are useful in this contest because they generate a single partition of the data in an attempt to recover the natural groups present in the data. Partitional methods are especially appropriate for the efficient representation and compression of large databases, as in the case of microcalcifications clustering [11].

If the clusters are compact and well separated, there is no uncertainty in assigning the objects to one cluster, but if the clusters are touching or overlapping, as is the case with microcalcifications clusters, the assignment of objects to clusters is difficult. So clustering methods based on fuzzy logic, according to which an object can belong to more than one cluster, are the better choice to handle the mammograms [22].

In the fuzzy clustering methods an object can belong to more than one cluster with a degree of membership continuously variable between 0 and 1. For ordinary clusters, the degree of membership for an object x is 1 if it belongs to the cluster and 0 if it does not; for the fuzzy clusters, larger is the membership degree to the clusters, greater will be the confidence level that the object belongs to that cluster [30-33]. The output of a fuzzy algorithm not only includes a partition but also additional information in the form of membership value.

The most known fuzzy clustering algorithm is the Fuzzy C- Means (FCM), fuzzy version of the K-means algorithm, which usually takes into account as feature only the position of microcalcification for the clustering process.

The method proposed here, instead, in addition to the spatial information used in the standard Fuzzy C-Means makes use of seven other features (in the following we refer to our method as Fuzzy C-Mean – With Features, FCM-WF) that add information about the structure of each single micro [34-36]. Three of these features take into account the geometric shape of an individual micro: area, perimeter, eccentricity. The other four take into account the pixels intensity variability of each micro: average, standard deviation, skewness and kurtosis of the intensity. In FCM-WF algorithm, clustering is performed not in Euclidean space but in multidimensional features space *F*.

**Figure 8:**
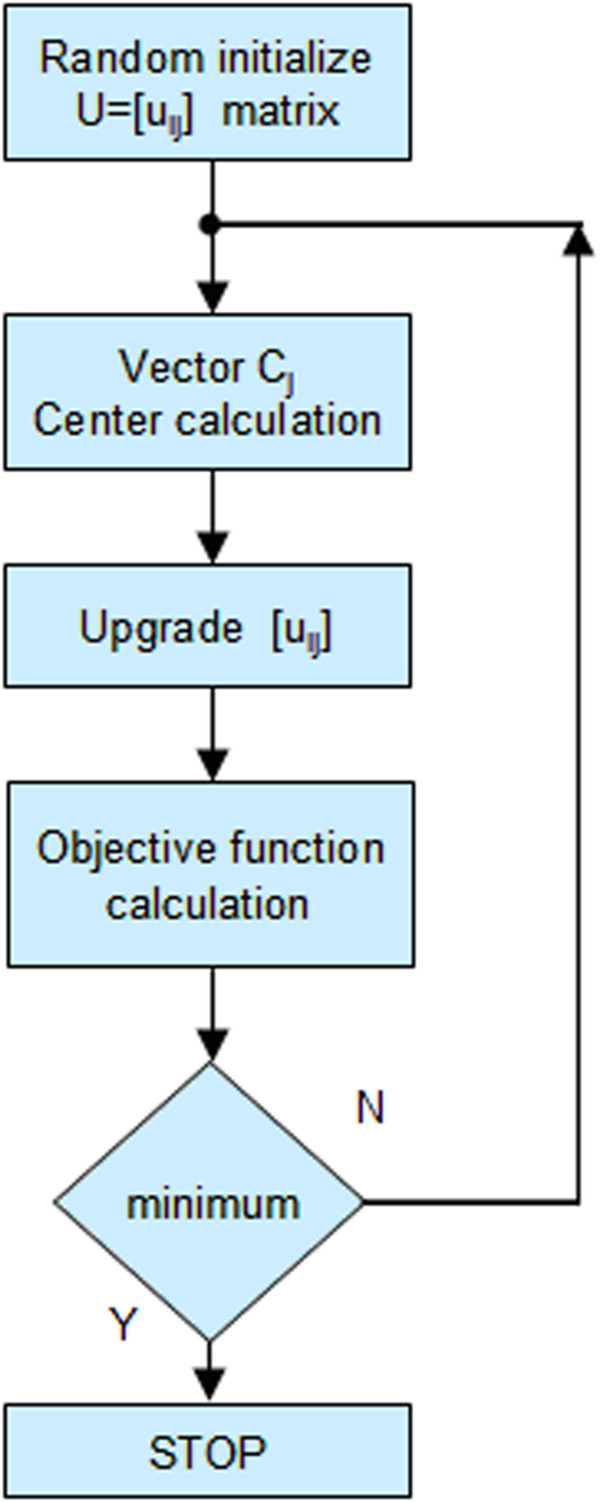
Minimization process of the objective function.

Given a set of N object, fuzzy partitioning in K clusters is carried out through an iterative optimization process that minimizes the following objective function

(2) $$J_{m}=\overset{N}{\underset{i=1}{\sum }}\overset{K}{\underset{j=1}{\sum }}u_{\mathrm{ij}}^{m}{∥X_{i}-C_{j}∥}^{2}$$

where X_i_ is the i-th object of the set X = {X_1_, X_2_, …X_N_} used to perform the clustering, with X_i_ ∈ *R*^*F*^ , i = 1 ….. N; similarly C_j_ is the j-th centroid of the set C = {C_1_, C_2_, …C_K_} with C_j_ ∈ *R*^*F*^, j = 1 ….. K. Finally u_ij_, is equal to the degrees of membership of the X_i_ object to the cluster C_j_. In Figure 8 we report the flow chart of the process. The initial values of the matrix U elements u_ij_ are randomly assigned; correspondently the objective function assumes the initial value J_0_. The iteration continues until reaching the absolute minimum of the objective function. The corresponding configuration of the clusters is considered as the best final result of the procedure used.

**Figure 9:**
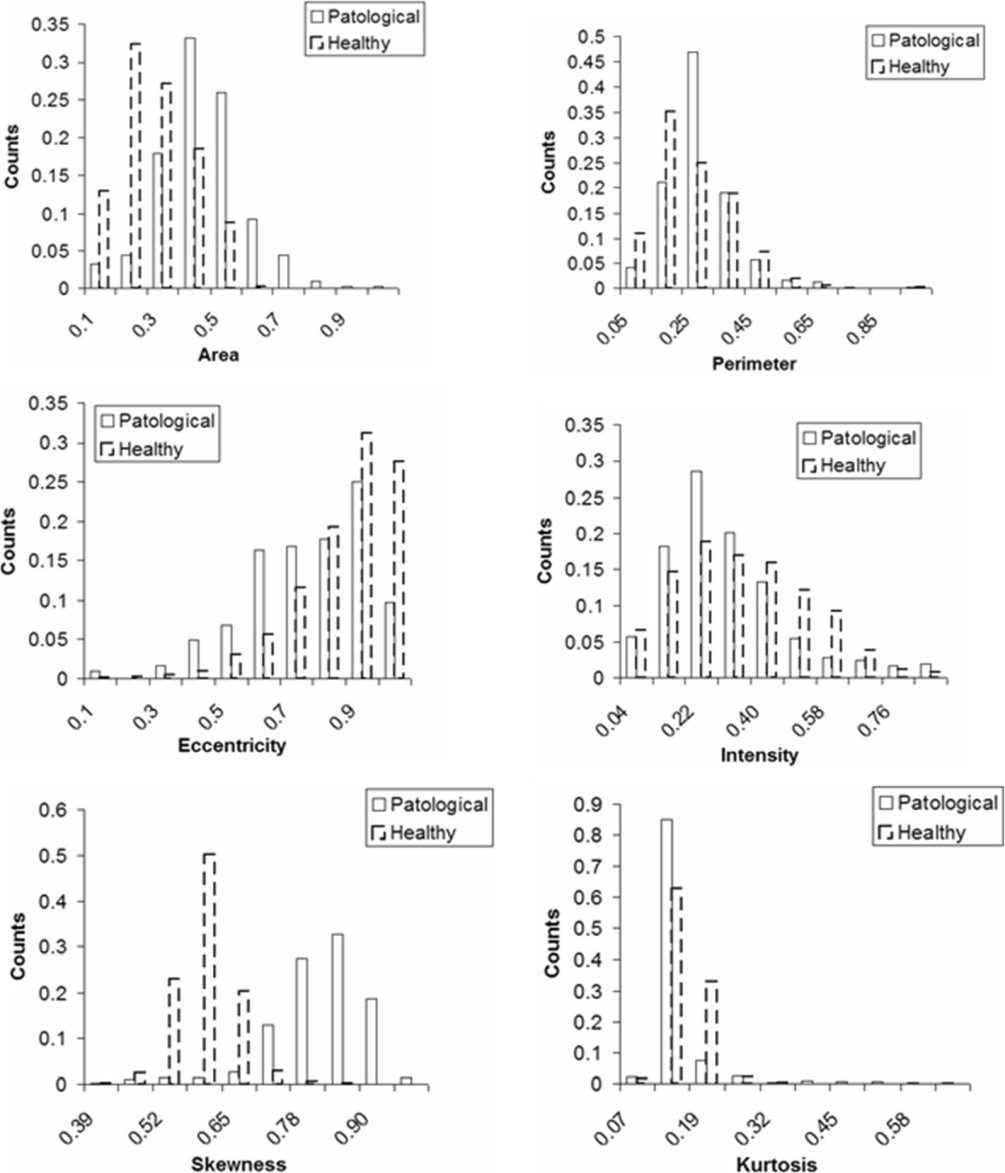
Features comparison: pathological/healthy.

It has been necessary to normalize all the features [37] because they are not dimensionless quantities, variable in a predetermined range; so, if x is a generic feature, its normalization in the range (0–1) can be carried out with the formula (3):

(3) $$x_{\text{norm}}=\frac{x-\text{min}}{\text{max}-\text{min}}$$

where, min = (*mean* - 4 *σ) and max = (*mean* + 4 *σ) and *mean* and σ are the mean value and the *standard deviation* of the feature x respectively. In Figure 9 the distributions of values for pathological and non-pathological segmented objects are compared for each feature.

### K Clusters Best Value

One of the problems in developing clustering algorithms is the *a priori* lack of knowledge of the number of clusters present in images. In Paragraph 5 we described the FCM-WF method, but we must remember that the number of clusters we are looking for is an input parameter to the clustering algorithm [34], K_ini_; it follows that it will be found always a number of clusters equal to the imposed value. For this reason we proposed a method to automatically determine the number of clusters present in an image based on the result of segmentation process: if the number of objects after the segmentation process is equal to N, since a disease cluster cannot have less than 3 micro, we can safely assume that the maximum number of clusters present in an image cannot exceed N/3. Moreover, we have a limit on cluster size because a pathological cluster has a maximum size of about 250 pixel (1 px = 0.07 mm).

If we start the clustering process on an image with the limit value K_ini_ = N/3 on the number of clusters, the minimization of the objective function will lead to a final configuration with N/3 clusters, some of which may have less than 3 micro (and they will be removed since they are not pathological) and other clusters with sizes that exceed the maximum size.

For this reason, at the end of the clustering process, the input value K_ini_ is reduced by N_down_ (number of clusters with less than 3 micro) and increased by N_up_ (number of clusters with spatial dimensions greater than the maximum), finding, at last, according to the equation 4, a final number W of residual clusters that may be less than K_ini_.

(4) $$W=K_{\text{ini}}-N_{\text{down}}+N_{\mathrm{up}}$$

Furthermore, since the clustering process starts with a random position of the centroids and with random values of the U matrix, the final number W of residues clusters could be dependent from the initial conditions. To get a more reliable estimate of W, we repeat the FCM-WF process several times starting with the same value of K_ini_ (for every image there is a different value of K_ini_, dependent from the number of segmented object present in the image), and calculate the average value $\overset{¯}{W}$ and the corresponding standard deviation σ_W_. Finally, to improve the significance of the average value $\overset{¯}{W}$, we introduce a rejection criterion: the elimination of the *W*_*i*_ values at a distance greater than 3* σ_W_ from $\overset{¯}{W}$. After this rejection, we recalculate the new $\overset{¯}{W}$ and σ_W_ values starting from a new value of K_ini_, according to the equation 5:

(5) $$K_{\text{ini}}=\overset{¯}{W}+3*\sigma_{W}$$

If the average value of W differs from K_ini_ for less than 3* σ_W_, the iterative process stops and a final run start with $K_{\text{ini}}=\overset{¯}{W}$.

**Figure 10:**
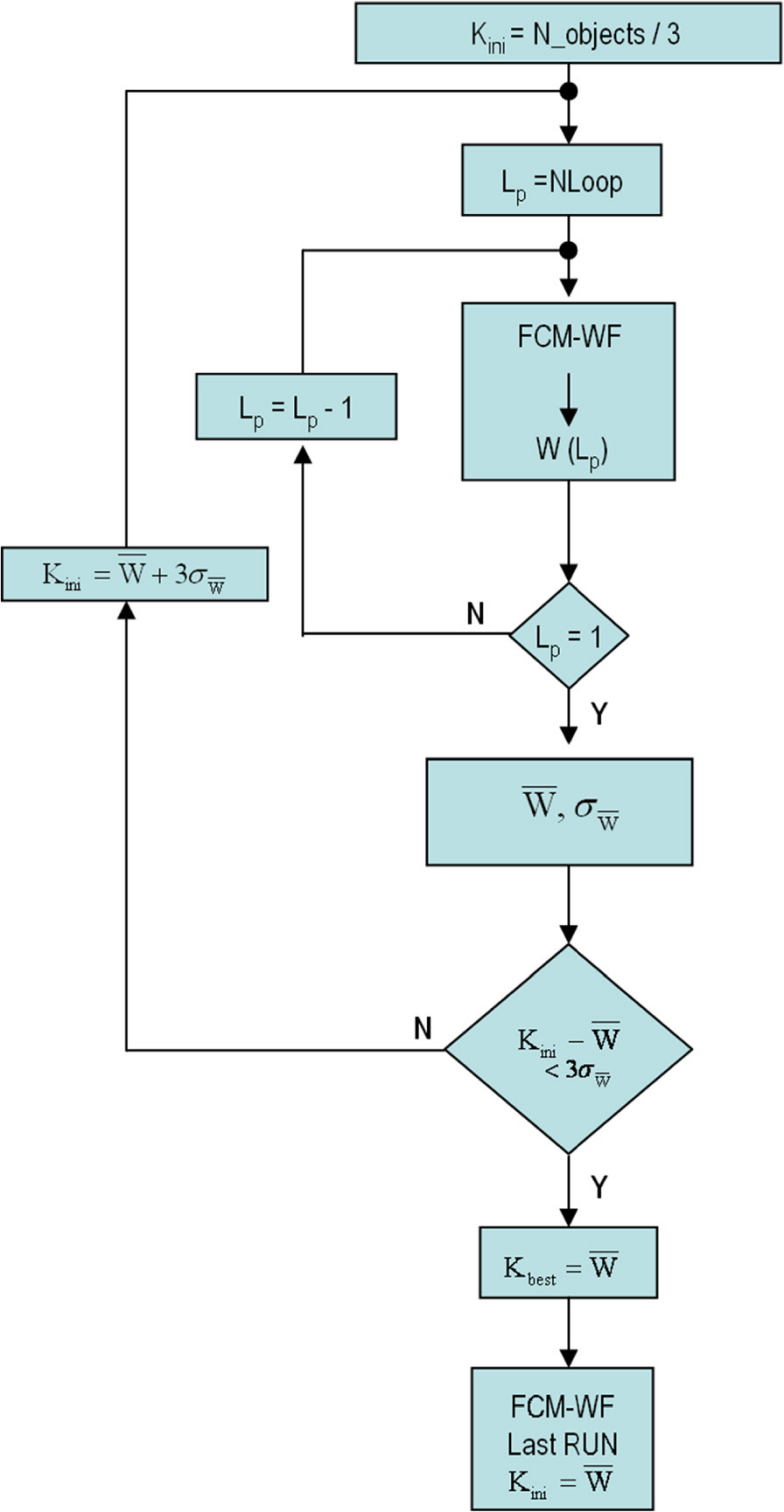
Iterative process to get the best value of cluster number.

The iterative process just described in the flow chart in Figure 10 that starts with K_ini_ = N/3 and repeat the process N_Loop_ times (in the example N_Loop_ = 50).

**Figure 11:**
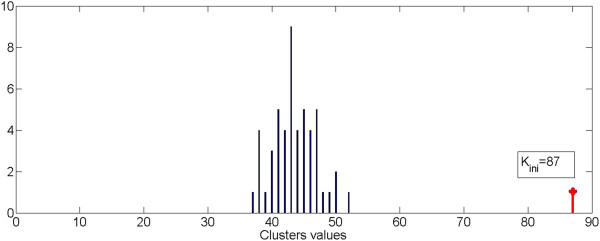
**Distribution of cluster values starting from K**_**ini**_ **= 87.**

**Figure 12:**
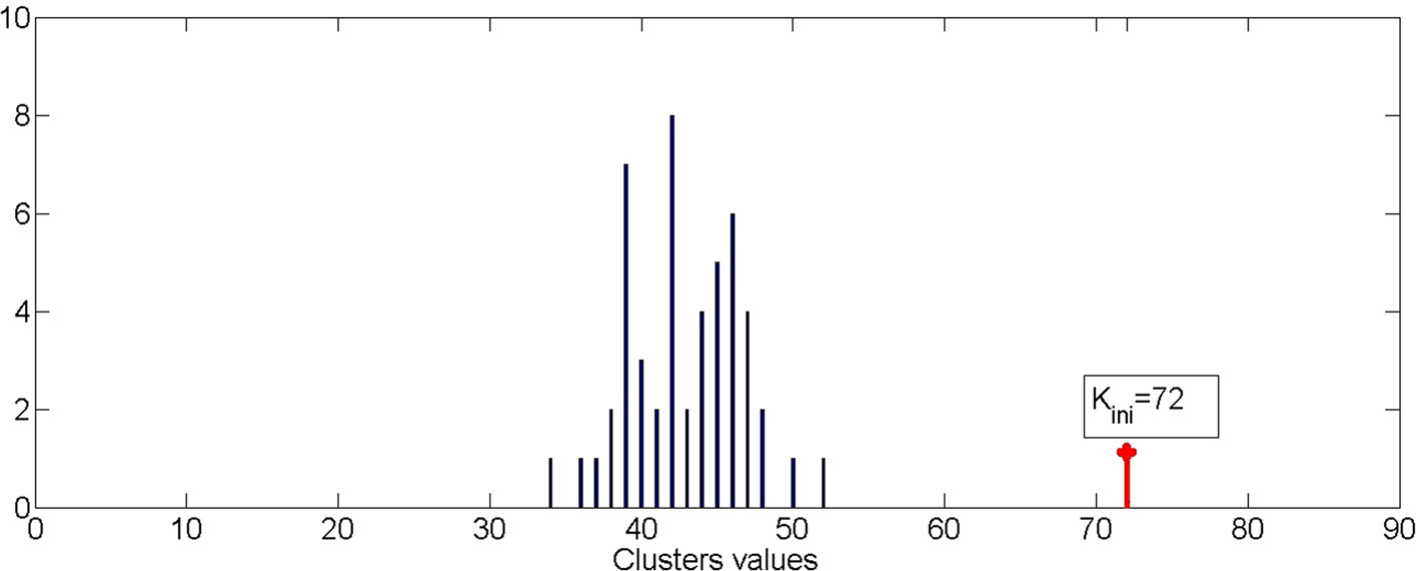
**Distribution of cluster values starting from K**_**ini**_ **= 72.**

Figure 11 shows the histogram of the values W(L_p_), with L_p_ = 1,…N_Loop_, obtained using an image with 261 segmented object and K_ini_ equal to 87. Figure 12 shows the histogram of the value W(L_p_) for the same image and positioned in the same range of variability as before but with a K_ini_ value equal to 72. This makes the process independent from the initial value of K_ini_, provided that this is greater than the number of residual final clusters.

**Figure 13:**
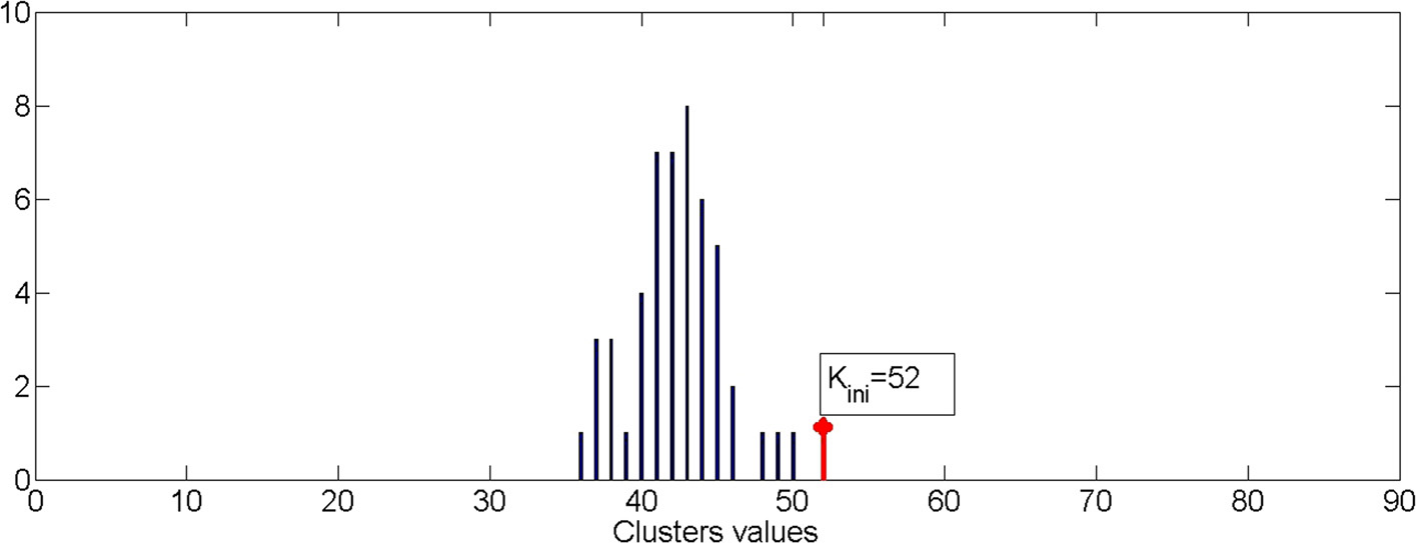
**Distribution of cluster values starting from K**_**ini**_ **= 52.**

Figure 13 shows the histogram of the value W(L_p_) obtained starting from an initial condition (value of K_ini_) closest to the final expected (stability condition).

## Results and discussions

The procedure described in the previous paragraphs was tested on 39 images of healthy patients belonging to a private database collected in the Policlinic Hospital of Palermo; every image has dimension of 4096 × 3328 and pixel spacing of 0.07 mm. The procedure was tested also on 20 images of the MIAS database; the size of all the images is 1024 × 1024 pixels.

Since in all the images the FCM-WF algorithm was able to recognize the clusters but the reconstruction turned out to be characterized by the lack of a few micros and the presence of some more objects (noise), we defined a *Merit Figure* as follows:

(6) $$F_{M}=\frac{N_{\text{ini}}-N_{\text{miss}}}{N_{\text{ini}}+N_{\text{noise}}}$$

In which is the number of micros belonging to the clusters, is the number of micros missing and is the number of noise objects added to the reconstructed cluster.

The performance of FCM-WF algorithm were compared with that of standard FCM. Indeed, to compare the results obtained by our proposed method with the existing ones, we used the following parameters:

TP = number of pathological micro correctly associated to the injected cluster;

FP = number of healthy segmented object erroneously associated to the injected cluster;

FN = number of pathological micro lost;

TN = number of healthy segmented object correctly associated to healthy clusters.

And evaluated also the following merit figures:

• Sensitivity = $\frac{\mathrm{TP}}{\left(\mathrm{TP}+\mathrm{FN}\right)}$

• Accuracy = $\frac{\mathrm{TP}+\mathrm{TN}}{\mathrm{TP}+\mathrm{TN}+\mathrm{FP}+\mathrm{FN}}$

• FP/Image = FP/tot. of segmented objects

• Precision = $\frac{\mathrm{TP}}{\mathrm{TP}+\mathrm{FP}}\;$

We reported in Table 1 the comparison between the performance of our method and that of the method already described with more details in paragraph 1, while the obtained results are presented in Section Results on private database and Section Results on MIAS database.

**Table 1:** Performance comparison of clustering methods for breast cancer detection

**Author**	**Clustering method**	**N. of mammograms (clusters)**	**Sensitivity**	**Accuracy**	**FP/Im**	**Precision**
**Nishikawa [**[12]**]**	**Spatial clustering**	**78 (41)**	**85%**	**-**	**-**	**-**
**Cihan [**[16]**]**	**Subtractive clustering**	**34 (72)**	**93%**	**-**	**-**	**-**
**Riyahi [**[17]**]**	**Wavelet transform and Fuzzy clustering**	**47 (47)**	**87%**	**-**	**0.5%**	**-**
**Cordella [**[18]**]**	**Graph-theoretical cluster analysis**	**40 (102)**	**94%**	**-**	**-**	**100%**
**Wang [**[19]**]**	**EFCM**	**180**	**-**	**99.7%**	**-**	**-**
**Quintanilla [**[20]**]**	**PFCM and ANN**	**-**	**98.2%**	**99.5%**	**-**	**-**
**Malar [**[21]**]**	**Wavelet and ELM**	**55**	**-**	**94%**		**-**
**Cheng [**[22]**]**	**FLSS**	**40 (105)**	**-**	**> 97%**	**14%**	**-**
**Our method (Private database)**	**FCM-WF**	**39 (39)**	**93%**	**95%**	**4%**	**62%**
**Our method (MIAS database)**	**FCM-WF**	**20(25)**	**82%**	**94%**	**4%**	**65%**

### Results on private database

Every image of the private database was injected with a pathological cluster with the mechanism described in Paragraph 4.1. The performance of the FCM-WF method has been evaluated by examining its capability of correctly recognize the injected clusters, i.e. by counting the number of microcalcifications correctly associated to the clusters. For this reason, as explained in Paragraph 4.1, it has been necessary to create a gold database in order to know all the truth and the details of microcalcifications clusters.

**Figure 14:**
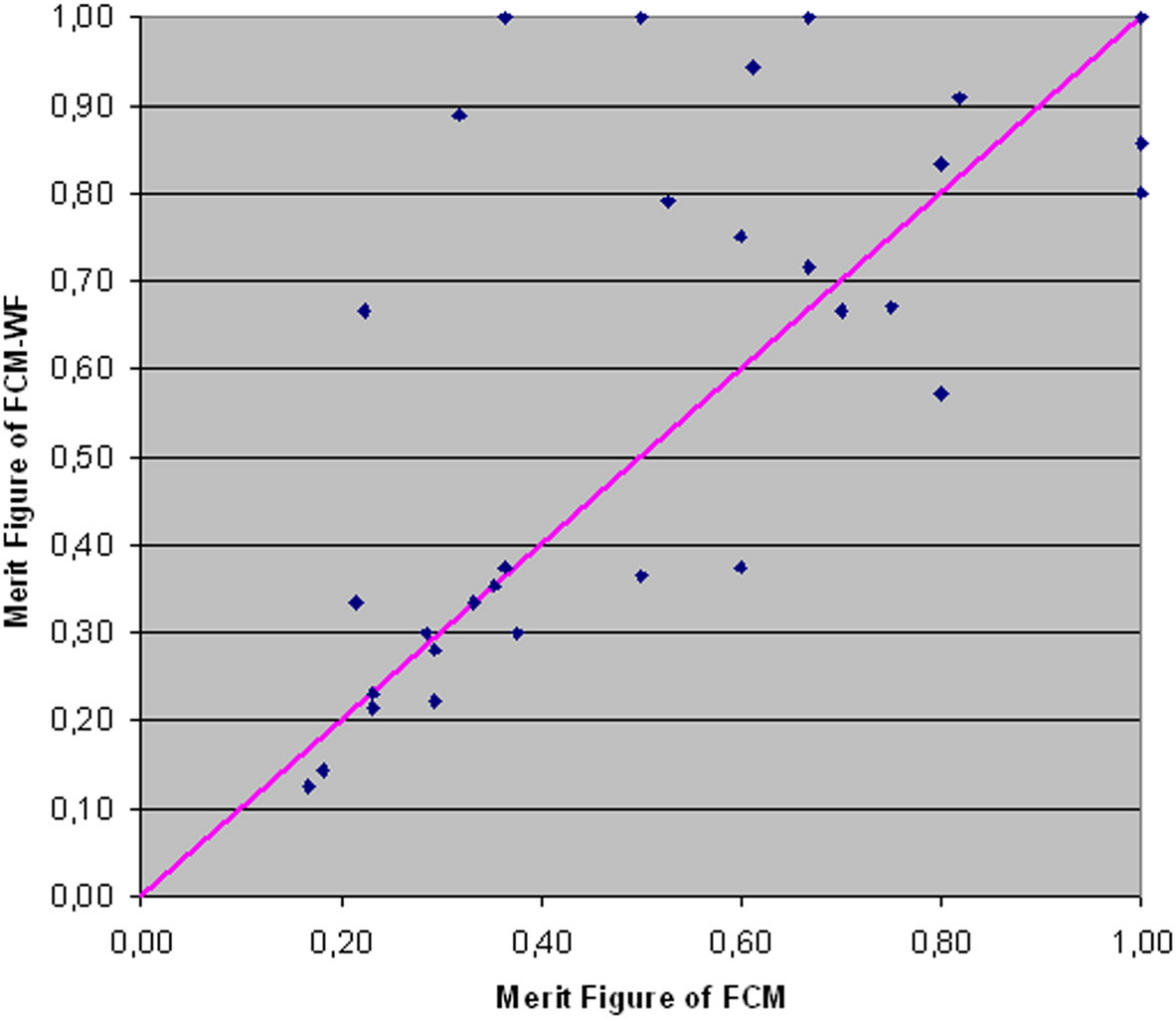
**Comparison between the *Merit Figures.***

**Figure 15:**
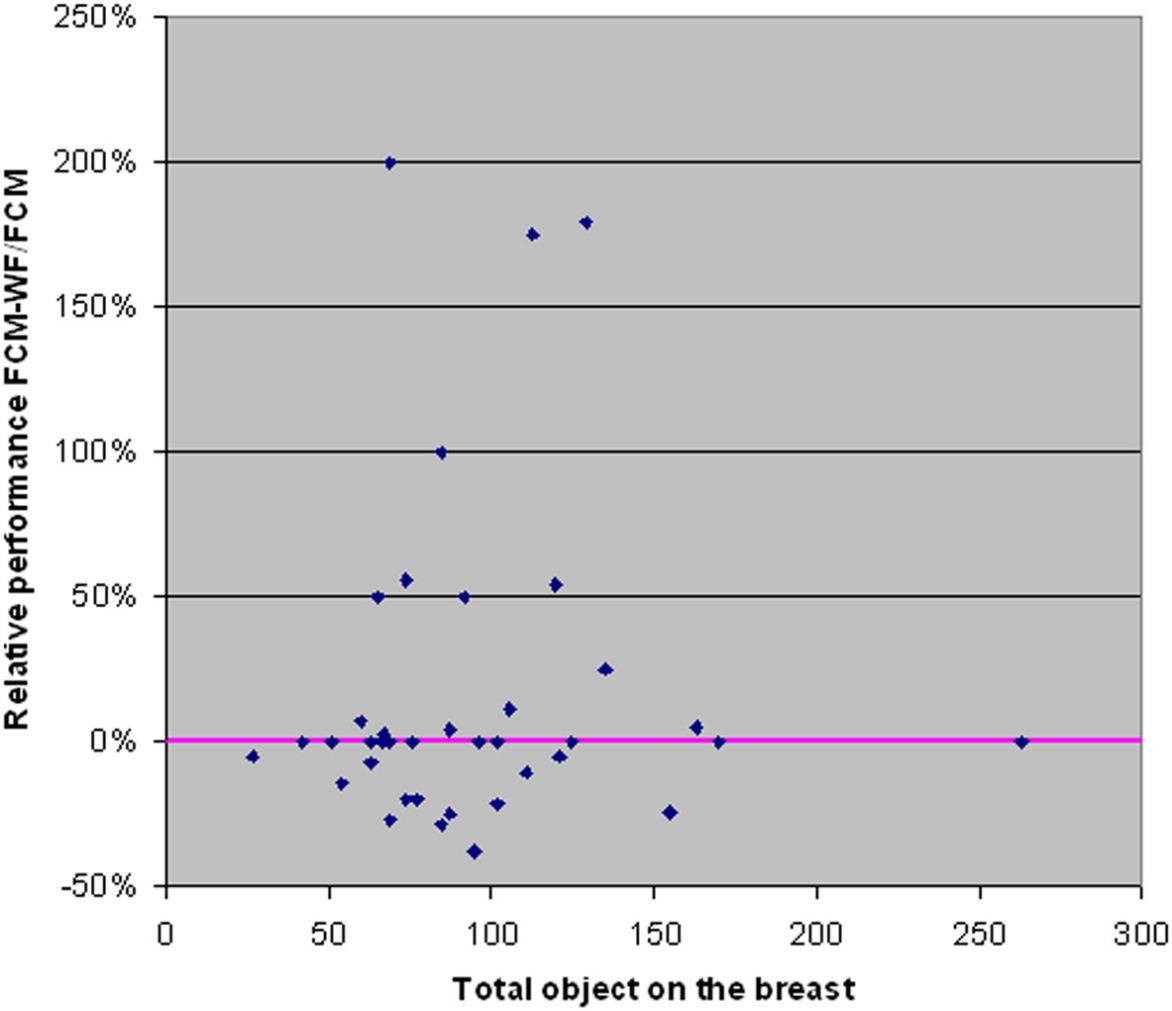
Relative performance improvement vs. total injected object.

In Figure 14 each point's coordinates represent the values of the *Merit Figure* obtained with the two methods: FCM (abscissa) and FCM-WF (ordinate); it is visible the improvement of the cases corresponding to the points above the diagonal line; the solid line corresponds to the cases that do not benefit in any way from the implemented method. Figure 15 highlights the relative performance (ratio) improvement as a function of the number of segmented objects in an image. If we denote by F_M1_ and F_M2_ the *Merit Figures* for the two methods FCM and FCM-WF respectively, the mean value of the two *Merit Figures* are 0.61 and 0.67 respectively, with an increase of about 10% in favor of the FCM-WF.

**Figure 16:**
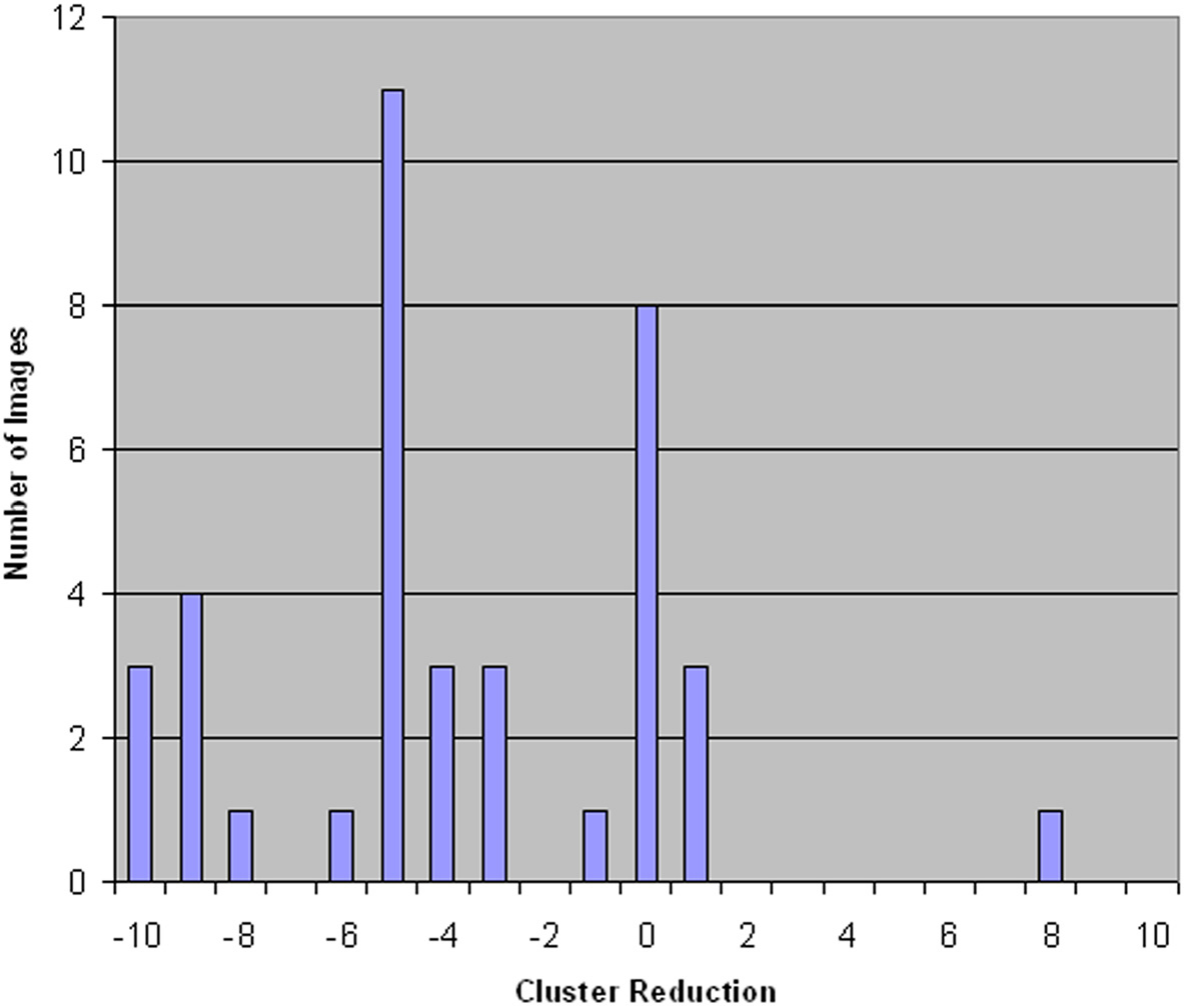
Residual clusters number reduction histogram.

**Figure 17:**
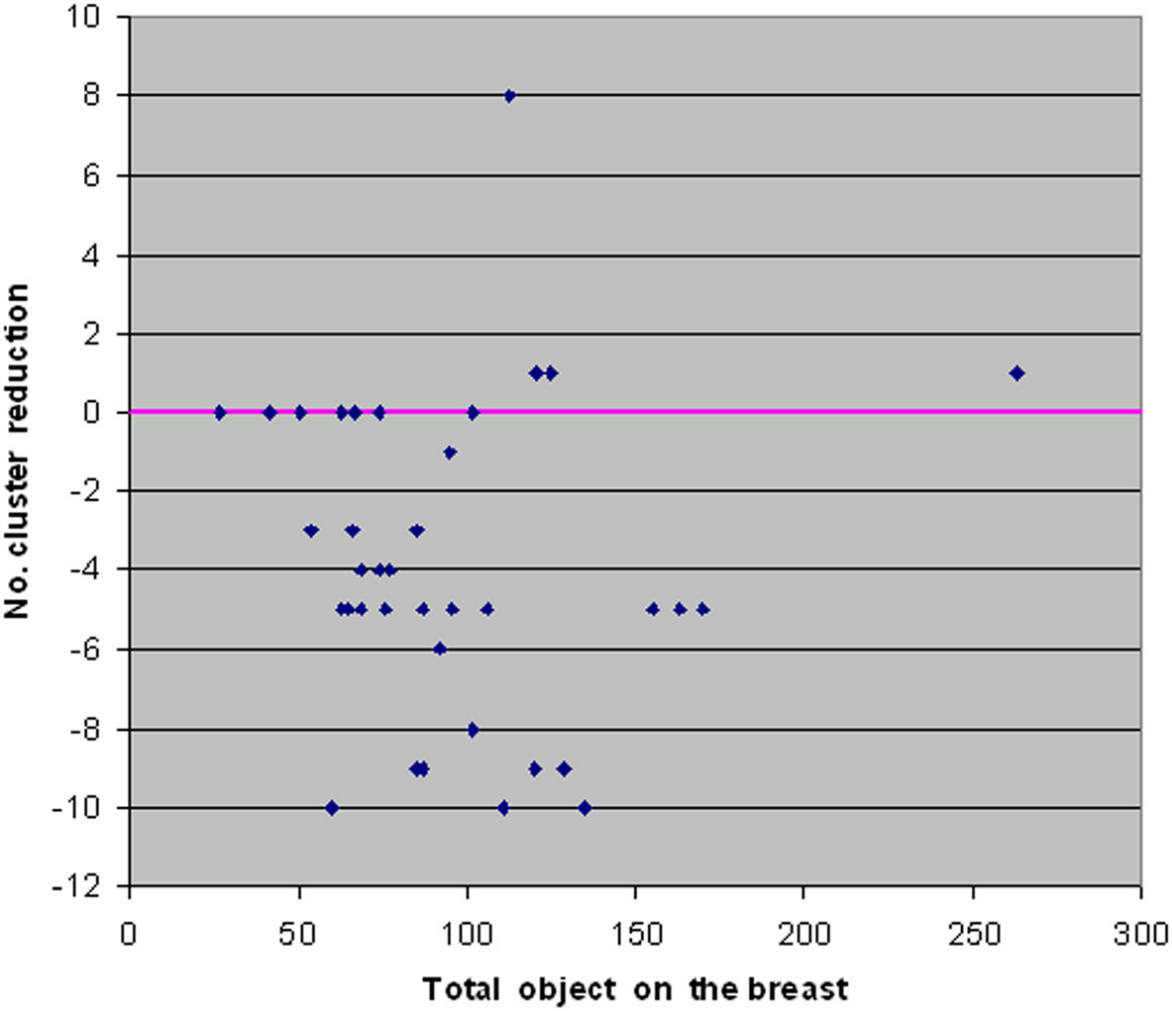
Residual clusters number reduction vs. total object on the breast image.

**Figure 18:**
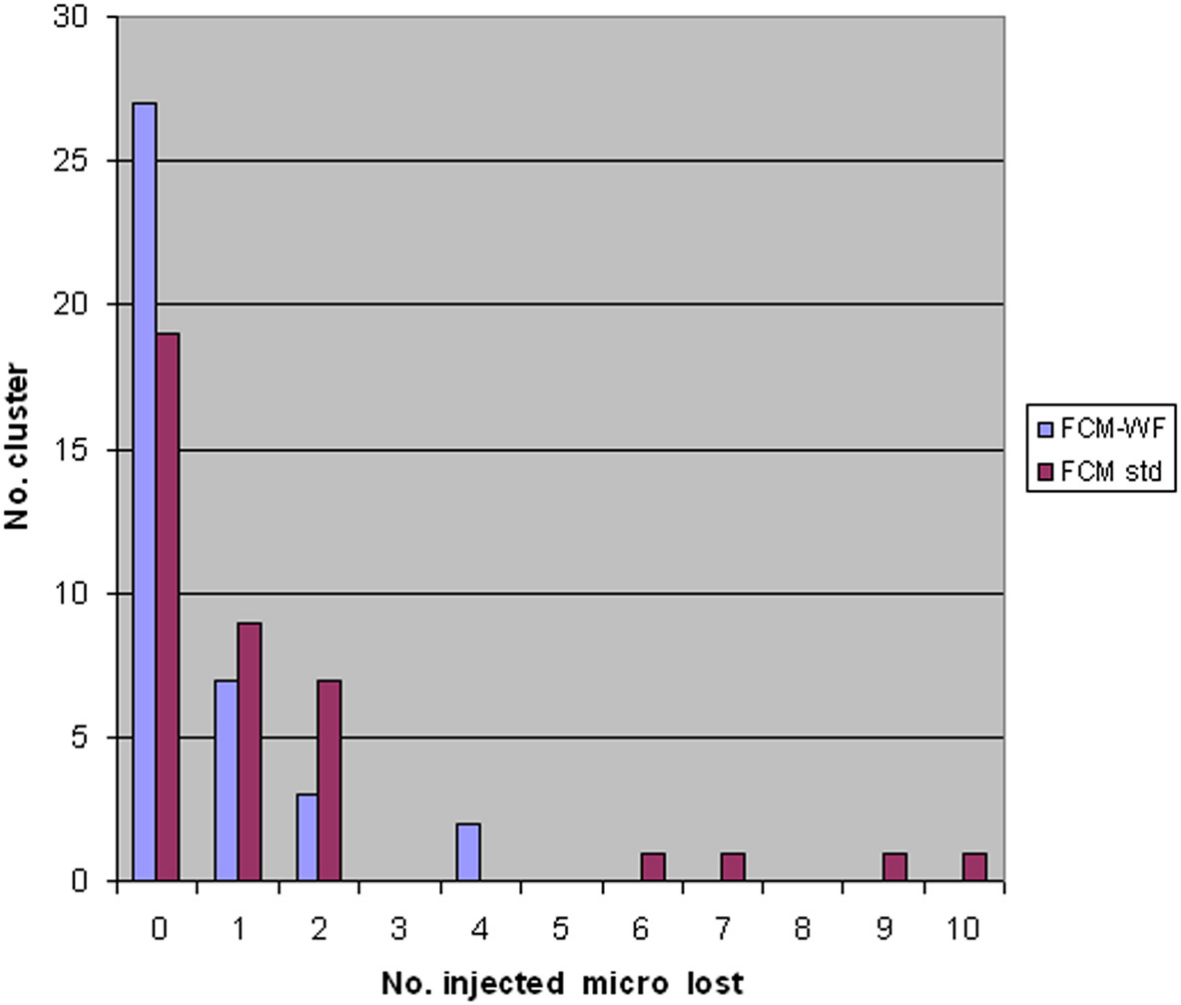
Number of lost injected micros.

For an expert system, such as a CAD, the clustering process is a preliminary step done before the classification stage. Therefore, while the presence of injected disease clusters is definitely positive for both methods FCM and FCM-WF because it does not alter the efficiency of disease detection, the presence of a large number of clusters in an image may have a negative effect by increasing the risk to accept an excessive number of false positives after the classification stage. Practically, with the method FCM-WF there has been a steady reduction in the number of residual clusters.In Figure 16 a histogram is displayed which illustrates the capacity of the FCM-WF method to reduce the number of residual clusters compared to the standard method FCM; in only one case there is a consistent increase in the number of residual clusters. In Figure 17 the reduction of the number of clusters is displayed as a function of the number of segmented objects present in the image before the clustering processes. It is easy to see from this graph that the reduction of the residual clusters does not depend on the number of objects initially present in the image. If we calculate the false positives number in the two methods FCM and FCM-WF for each of the 39 images used, we obtain a false positives average number reduction equal to about 22%.The histogram in Figure 18 illustrates the ability of the two methods to maintain the pathological cluster: the results point out that FCM-WF achieves a better performance with respect to the conventional method. In particular, among the injected clusters, 70% remain unaffected if the reconstruction is performed with the FCM-WF.

The method was also evaluated in terms of Sensitivity, Accuracy, FP/image and Precision obtaining the following results:

• Sensitivity = $\frac{\mathrm{TP}}{\left(\mathrm{TP}+\mathrm{FN}\right)}=93\%$

• Accuracy = $\frac{\mathrm{TP}+\mathrm{TN}}{\mathrm{TP}+\mathrm{TN}+\mathrm{FP}+\mathrm{FN}}=95\%$

• FP/Image = FP/tot. of segmented objects = 4%

• Precision = $\frac{\mathrm{TP}}{\mathrm{TP}+\mathrm{FP}}=62\%$

### Results on MIAS database

The FCM-WF method has been tested also on 20 images belonging to the mini-MIAS database. As explained in Section The MIAS database, the database provide only the centre locations and radii of clusters, and since we need to know even position and number of every microcalcification belonging to it, we considered as “truth” about pathological microcalcifications the objects found inside the indicated circle after the segmentation process.

For this database we defined also a “segmentation efficiency” as the number of cluster found in the images after the segmentation process, which is equal to 80%.

**Figure 19:**
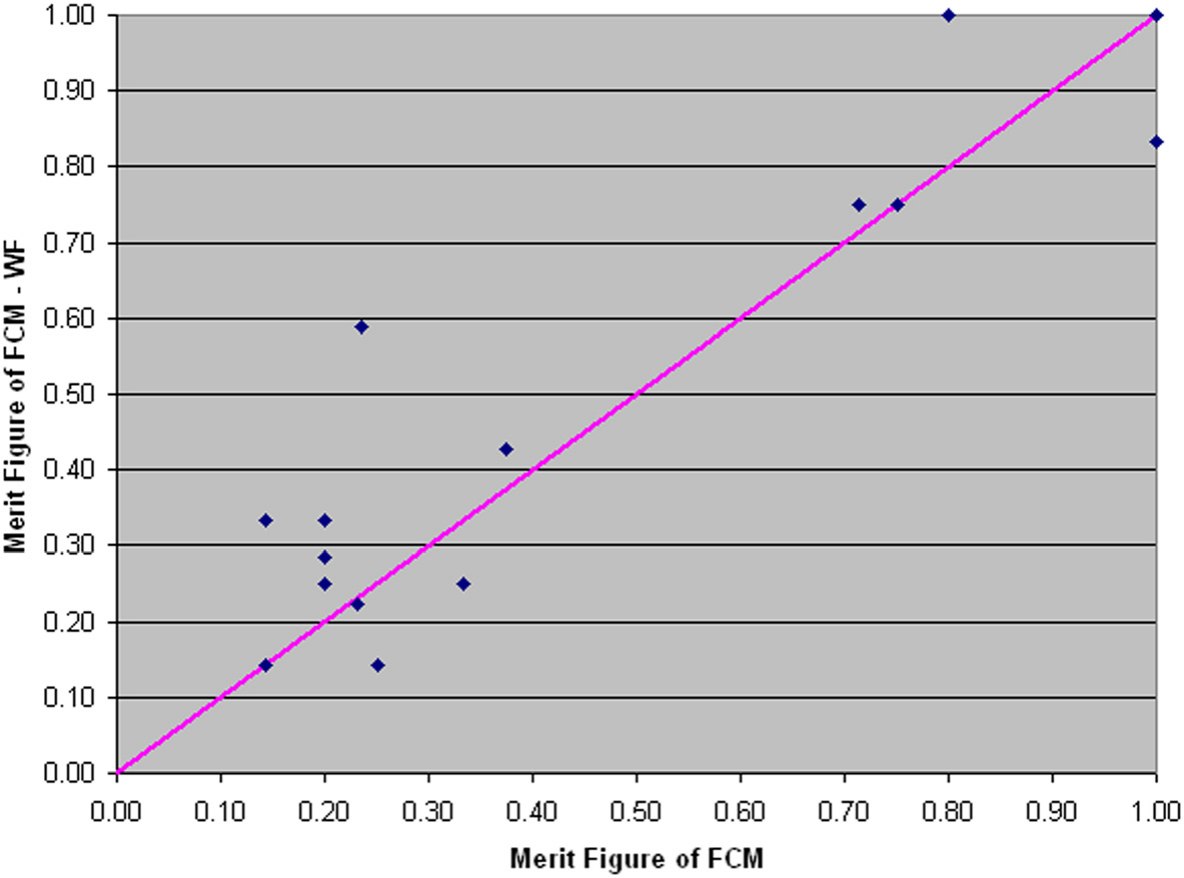
**Comparison between the *Merit Figures.***

**Figure 20:**
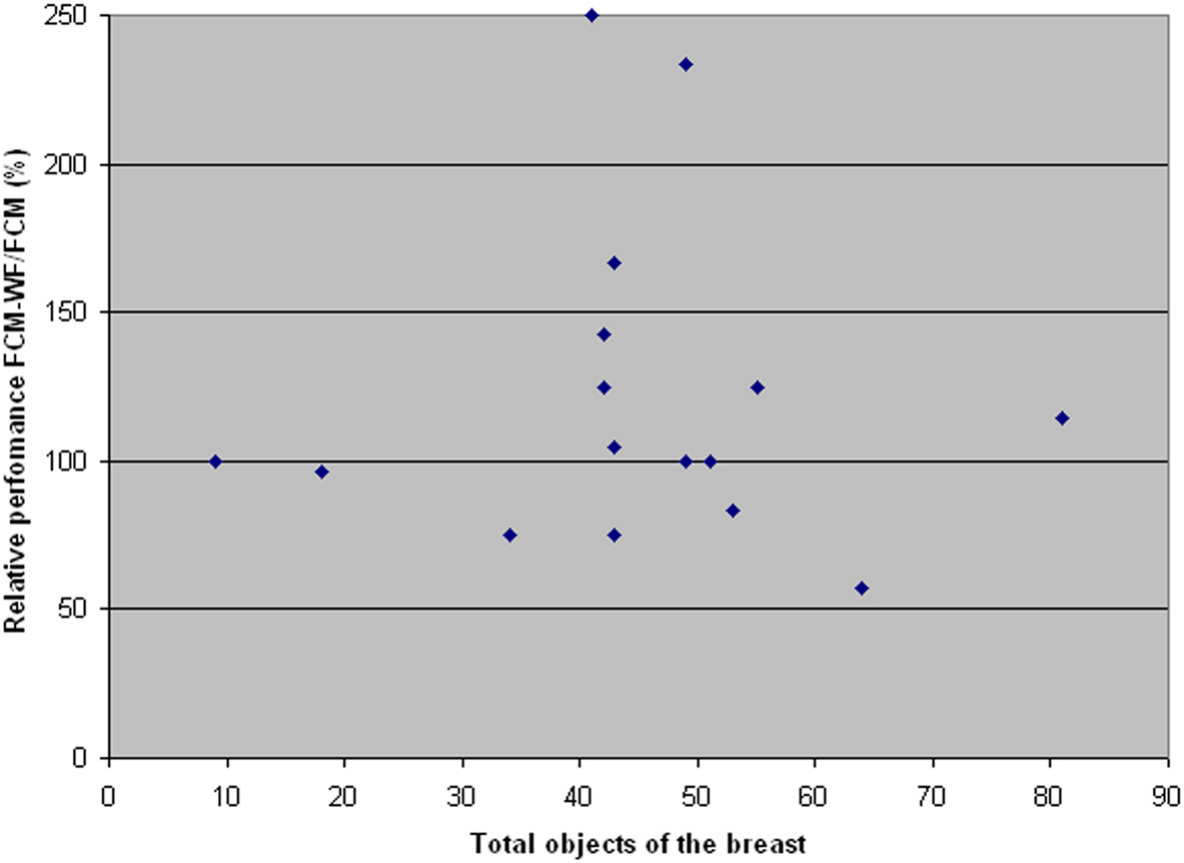
Relative performance improvement vs. total injected object.

In Figure 19 each point's coordinates represent the values of the *Merit Figure* obtained with the two methods: FCM (abscissa) and FCM-WF (ordinate); even for this database it is visible the improvement of the cases corresponding to the points above the diagonal line. Figure 20 highlights the relative performance (ratio) improvement as a function of the number of segmented objects in an image. If we denote by F_M1_ and F_M2_ the *Merit Figures* for the two methods FCM and FCM-WF respectively, the mean value of the two *Merit Figures* are 0.45 and 0.50 respectively, with an increase of 5% in favor of the FCM-WF.

If we calculate the false positives number in the two methods FCM and FCM-WF for every image of the MIAS database used, we obtain a false positives average number reduction equal to about 10%.

The method was also evaluated in terms of Sensitivity, Accuracy, FP/image and Precision obtaining the following results:

• Sensitivity = $\frac{\mathrm{TP}}{\left(\mathrm{TP}+\mathrm{FN}\right)}=82\%$

• Accuracy = $\frac{\mathrm{TP}+\mathrm{TN}}{\mathrm{TP}+\mathrm{TN}+\mathrm{FP}+\mathrm{FN}}=94\%$

• FP/tot. of segmented objects = 4 %

• Precision = $\frac{\mathrm{TP}}{\mathrm{TP}+\mathrm{FP}}=65\%$

## Conclusions

In this paper we presented a clustering method for microcalcifications based on fuzzy logic. This method, called Fuzzy C-Mean With Features (FCM-WF) allows microcalcifications clustering not only according to their distance but also to their relevant features.

The method was tested on a database of simulated images obtained by injecting a pathological cluster on healthy images: in this way we know the “truth” about the exact position of a cluster and the number of microcalcifications belonging to it.

Thanks to this database and to the informations contained in it regarding every single microcalcification, we tested the developed clustering method with great accuracy: actually, for every cluster, we verified the difference between the desired and obtained result with the *Merit Figure* of equation 6. In particular, we verified that 70% of the injected clusters remained unaffected if the reconstruction is performed with the FCM-WF.

Moreover, the automatic determination of the number of clusters in an image result in further improvements of the overall performance of the FCM-WF method over the standard FCM.

Finally, we want to put in evidence in this paper that the technique of placing the micro image in a healthy described here is not equivalent to a classic simulation process by injection of small spots of pixels of variable amplitude and positions randomly, but consists in the placing of real pathological cluster that retain and transfer the original information through the features used here, allowing us to consider the database simulated as a real database of reported image (Gold Standard Database).

In order to provide strong justification for the effectiveness of our work, we applied the FCM-WF algorithm even on the publicly available MIAS database. However, since to test the method we have to know position and number of microcalcifications belonging to the clusters, we didn’t use all images of the database but only the images in which centre locations and radii of clusters are known. We considered as “truth” about pathological microcalcifications the objects found inside the indicated circle after the segmentation process, so we defined a “segmentation efficiency” as the number of cluster found in the images after the segmentation process, which is equal to 80%. For this database we obtained an increase of 5% of the Merit Figure of the FCM-WF compared to that of FCM and a false positives average number reduction equal to about 10%.

## Competing interests

We haven’t received in the past five years reimbursements, fees, funding, or salary from an organization that may in any way gain or lose financially from the publication of this manuscript, either now or in the future. We don’t hold any stocks or shares in an organization that may in any way gain or lose financially from the publication of this manuscript, either now or in the future. We don’t hold or we aren’t currently applying for any patents relating to the content of the manuscript. We haven’t received reimbursements, fees, funding, or salary from an organization that holds or has applied for patents relating to the content of the manuscript. We haven’t any other financial competing interests. There aren’t any non-financial competing interests (political, personal, religious, ideological, academic, intellectual, commercial or any other) to declare in relation to this manuscript.

## Authors’ contributions

LV conceived of the study, carried out the Clustering algorithm implementation and determination of K best value, performed the statistical analysis and drafted the manuscript. DC carried out the microcalcifications segmentation, the gold database creation and injection process. FF and GR conceived of the study, participated in the design and coordination of the manuscript, and helped to its draft. All authors read and approved the final manuscript.

## Pre-publication history

The pre-publication history for this paper can be accessed here:

http://www.biomedcentral.com/1471-2342/14/23/prepub

## References

[B1] Ciatto S Cascio D Fauci F Magro R Raso G Ienzi R Martinelli F Simone MV Computer-Assisted Diagnosis (CAD) in Mammography: Comparison of Diagnostic Accuracy of a New Algorithm (Cyclopus^®^, Medicad) with Two Commercial Systems Radiol Med 2009 114 626 635 10.1007/s11547-009-0396-4 19444587 10.1007/s11547-009-0396-4

[B2] Cascio D Fauci F Iacomi M Raso G Magro R Castrogiovanni D Filosto G Ienzi R Vasile MS Computer-aided diagnosis in digital mammography: comparison of two commercial systems Imaging in Medicine 2014 6 1 13 20

[B3] Karssemeijer N Hendriks JHCL Computer-assisted reading of mammograms Eur Radiol 1997 7 743 748 9166576 10.1007/BF02742937

[B4] McLoughlin KJ Bones PJ Karssemeijer N Noise equalization for detection of microcalcification clusters in direct digital mammogram images IEEE Trans Med Imag 2004 23 3 313 320 10.1109/TMI.2004.824240 15027524

[B5] Fu JC Lee SK Wong STC Yeh JY Wang AH Wu HK Image segmentation feature selection and pattern classification for mammographic microcalcifications Comput Med Imaging Graph 2005 29 419 429 16002263 10.1016/j.compmedimag.2005.03.002

[B6] Halkiotis S Botsis T Rangoussi M Automatic detection of clustered microcalcifications in digital mammograms using mathematical morphology and neural networks Signal Process 2007 87 1559 1568

[B7] Vedkamp WJH Karssemeijer N Automated classification of clustered microcalcifications into malignant and benign types Med Phys 2000 27 11 2600 2608 11128313 10.1118/1.1318221

[B8] Cascio D Magro R Fauci F Iacomi M Raso G Automatic detection of lung nodules in CT datasets based on stable 3D mass-spring models Comput Biol Med 2012 42 11 1098 1109 23020972 10.1016/j.compbiomed.2012.09.002

[B9] Cascio D Magro R Fauci F Iacomi M Raso G Automatic detection of lung nodules in low-dose computed tomography Comput Assist Radiol Surg 2007 2 supplement 1 357 360

[B10] Wei L Yang Y Nishikawa RN Jiang Y A study on several machine-learning methods for classification of malignant and benign clustered microcalcifications IEEE Trans Med Imag 2005 24 3 371 380 10.1109/tmi.2004.842457 15754987

[B11] Jain AK Dubes RC Algorithms for clustering data 1988 Upper Saddle River, NJ, USA: Prentice Hall 55 143

[B12] Nishikawa RM Giger ML Doi K Vyborny CJ Schmidt RA Computer-aided detection of clustered microcalcifications: an improved method for grouping detected signals Med Phys 1993 20 6 1661 1666 8309438 10.1118/1.596952

[B13] Estevez L Kehtarnavaz N Wendt R Interactive selective and adaptive clustering for detection of microcalcifications in mammograms Digit Signal Process 1996 6 224 232

[B14] Mao F Zhang Y Song D Qian W Clarke LP An improved method of region grouping for microcalcification detection in digital mammograms” Proc of IEEE 1998 20 2 740 743 10.1016/s0895-6111(02)00045-9 12453502

[B15] Arodz T Kurdziel M Popiela TJ Sevre E Yuen D Detection of clustered microcalcifications in small field digital mammography” Comput Methods Prog Biomed 2006 81 56 65 10.1016/j.cmpb.2005.10.002 16310282

[B16] Cihan IK Senel HG An application of topological median filter on detection and clustering of microcalcifications in digital mammograms IEEE 2006 10 1136 1139

[B17] Riyahi-Alam N Ahmadian A Nasehi Tehrani J Guiti M Oghabian MA Segmentation of suspicious clustered microcalcifications on digital mammograms: using fuzzy logic and wavelet7th International Conference on Signal Processing Proceedings, ICSP 2004 3 2228 2230

[B18] Cordella LP Percannella G Sansone C Vento M A graph-theoretical clustering method for detecting clusters of microcalcifications in mammographic images Proceedings of the IEEE CBMS 2005 15 20

[B19] Wang Y Shi H Ma S A new approach to the detection of lesions in mammography using fuzzy clustering J Int Med Res 2011 39 2256 2263 22289541 10.1177/147323001103900622

[B20] Quintanilla-Dominguez J Ojeda-Magana B Marcano-Cedeno A Barròn-Adame JM Vega-Corona A Andina D Automatic detection of microcalcifications in ROI images based on PCFM And ANN Int J Intell Comput Med Sci Image Process 2013 5 2 161 174

[B21] Malar E Kandaswamy A Chakravarthy D Giri DA A novel approach for detection and classification of mammographic microcalcifications using wavelet analysis and extreme learning machine Comput Biol Med 2012 42 898 905 22871899 10.1016/j.compbiomed.2012.07.001

[B22] Cheng HD Wang J Shi X Microcalcification detection using fuzzy logic and scale space approaches Pattern Recogn 2004 37 363 375

[B23] Fauci F Bagnasco S Bellotti R Cascio D Cheran SC De Carlo F De Nunzio G Fantacci ME Forni G Lauria A Lopez Torres E Magro R Masala GL Oliva P Quarta M Raso G Retico A Tangaro S Mammogram segmentation by contour searching and massive lesion classification with neural network IEEE Nucl Sci Symp Conf R 2004 5 2695 2699

[B24] Fauci F Cascio D La Manna A Magro R Raso G Vasile M Iacomi M A fourier-based algorithm for micro-calcification enhancement in mammographic images IEEE Nuclear Science Symposium Conference Record, article number 4774254 2008 4388 4391

[B25] Liu J Chen J Liu X Chun L Tang J Deng Y Mass segmentation using a combined method for cancer detection BMC Syst Biol 2011 5 SUPPL. 3 art. no. S6 10.1186/1752-0509-5-S3-S6 PMC3287574 22784625

[B26] Iacomi M Cascio D Fauci F Raso G Mammographic images segmentation based on chaotic map clustering algorithm BMC Med Imaging 2014 14 1 12 24666766 10.1186/1471-2342-14-12 PMC3987162

[B27] Marr D Vision: A computational investigation into the human representation and processing of visual information: San Francisco 1982

[B28] Tangaro S Bellotti R De Carlo F Gargano G Lattanzio E Monno P Massacra R Delogu P Fantacci ME Retico A Mazzocchi M Bagnasco S Cerello P Cheran SC Lopez Torres E Zanon E Lauria A Sodano A Cascio D Fauci F Magro R Raso G Ienzi R Bottigli U Masala GL Oliva P Meloni G Caricato AP Cataldo R MAGIC-5: an Italian mammographic database of digitized images for research La Radiologia Medica 2008 113 4 477 485 18536871 10.1007/s11547-008-0282-5

[B29] Youn H Han Jong C Cho Min K Jang SY Kim HK Kim JH Tanguay J Cunningham IA Numerical generation of digital mammograms considering imaging characteristics of an imager Nucl Instrum Meth A 2011 652 810 814

[B30] Berthold M Hand DJ Intelligent data analysis 1999 Book: Springer 321 351

[B31] Vivona L Cascio D Magro R Fauci F Raso G A fuzzy logic C-means clustering algorithm to enhance microcalcifications clusters in digital mammograms IEEE Nucl Sci Symp Med Imaging Conf 2012 3048 3050 art. No. 6152551

[B32] Bellotti R De Carlo F Gargano G Tangaro S Cascio D Catanzariti E Cerello P Cheran SC Delogu P De Mitri I Fulcheri C Grosso D Retico A Squarcia S Tommasi E Golosio B A CAD system for nodule detection in low-dose lung CTs based on region growing and a new active contour model Med Phys 2007 34 12 4901 4910 18196815 10.1118/1.2804720

[B33] Rusina R Kukal J Bělíček T Buncová M Matěj R Use of fuzzy edge single-photon emission computed tomography analysis in definite Alzheimer's disease - a retrospective study BMC Med Imaging 2010 10 , art. no. 20 10.1186/1471-2342-10-20 PMC2939533 20809946

[B34] Masala GL Golosio B Oliva P Cascio D Fauci F Tangaro S Quarta M Cheran SC Lopez Torres E Classifiers trained on dissimilarity representation of medical pattern: a comparative study” Nuovo Cimento della Società Italiana di Fisica C 2005 28 6 905 912

[B35] Masala GL Tangaro S Golosio B Oliva P Stumbo S Bellotti R De Carlo F Gargano G Cascio D Fauci F Magro R Raso G Bottigli U Cgincarini A De Mitri I De Nunzio G Gori I Retico A Cerello P Cheran SC Fulcheri C Lopez Torres E Comparative study of feature classification methods for mass lesion recognition in digiteized mammograms” Nuovo Cimento C, Geophysics and Space Physics 2007 30 3 305 316

[B36] McKeen-Polizzotti L Henderson KM Oztan B Bilgin CC Yener B Plopper GE Quantitative metric profiles capture three-dimensional temporospatial architecture to discriminate cellular functional states” BMC Med Imaging 2011 11, art. no. 11 10.1186/1471-2342-11-11 PMC3125246 21599975

[B37] Kim SY Lee JW Bae JS Effect of data normalization on fuzzy clustering of DNA microarray data BMC Bioinformatics 2006 7, art. no. 134 10.1186/1471-2105-7-134 PMC1431564 16533412

